# Meteorin Is a Novel Therapeutic Target for Wet Age-Related Macular Degeneration

**DOI:** 10.3390/jcm10132973

**Published:** 2021-07-02

**Authors:** Kimberley Delaunay, Alexandre Sellam, Virginie Dinet, Alexandre Moulin, Min Zhao, Emmanuelle Gelizé, Jérémie Canonica, Marie-Christine Naud, Patricia Crisanti-Lassiaz, Francine Behar-Cohen

**Affiliations:** 1 Centre de Recherche des Cordeliers, Sorbonne Université, Université de Paris, INSERM, From Physiopathology of Retinal Diseases to Clinical Advances, 75006 Paris, France; kimberley.delaunay@etu.u-paris.fr (K.D.); alexandresellam@gmail.com (A.S.); virginie.dinet@inserm.fr (V.D.); elodiecn@gmail.com (M.Z.); emma-nuelle.gelize@gmail.com (E.G.); jerem.canonica@gmail.com (J.C.); marie-christine.naud@crc.jussieu.fr (M.-C.N.); patricia.lassiaz@gmail.com (P.C.-L.); 2 Biology of Cardiovascular Diseases, INSERM U1034, Pessac, Université de Bordeaux, 33000 Bordeaux, France; 3 Department of Ophthalmology, University of Lausanne, Jules Gonin Eye Hospital, Fondation Asile des Aveugles, 1000 Lausanne, Switzerland; alexandre.moulin@fa2.ch; 4 Hôpital Cochin Ophthalmopole, Assistance Publique—Hôpitaux de Paris, 75014 Paris, France; 5 INSERM UMR_S 1138, Team 17: From Physiopathology of Retinal Diseases to Clinical Advances, Centre de Recherche des Cordeliers, 75006 Paris, France

**Keywords:** retina, choroidal neovascularization, angiogenesis, meteorin, therapeutic innovation

## Abstract

The aim of this study was to evaluate the potential anti-angiogenic effect of MTRN (meteorin) in the laser-induced CNV rat model and explore its mechanisms of action. MTRN, thrompospondin-1, glial cell markers (GFAP, vimentin), and phalloidin were immuno-stained in non-human primate flat-mounted retinas and human retina cross sections. The effect of MTRN at different doses and time points was evaluated on laser-induced CNV at 14 days using in vivo fluorescein angiography and ex vivo quantification of CNV. A pan transcriptomic analysis of the retina and the RPE/choroid complex was used to explore MTRN effects mechanisms. In human retina, MTRN is enriched in the macula, expressed in and secreted by glial cells, and located in photoreceptor cells, including in nuclear bodies. Intravitreal MTRN administered preventively reduced CNV angiographic scores and CNV size in a dose-dependent manner. The highest dose, administered at day 7, also reduced CNV. MTRN, which is regulated by mineralocorticoid receptor modulators in the rat retina, regulates pathways associated with angiogenesis, oxidative stress, and neuroprotection. MTRN is a potential novel therapeutic candidate protein for wet AMD.

## 1. Introduction

Age-Related Macular Degeneration (AMD) is the most common retinal disease, affecting 200 million individuals over 60 years old world-wide [1]. The “wet” form of AMD, also called neovascular or exudative AMD, represents approximately 50% of the cases, and is characterized by the growth of new abnormal blood vessels from the choroid into the retina, called choroidal neovascularization (CNV), causing macular edema, which is associated with rapid and severe vision loss. Anti-VEGF (Vascular Endothelial Growth Factor) intraocular injections stabilize vision in 80% of the cases at the cost of multiple injections [2,3]. However, after one year of optimal treatment regimens, only 40% of patients respond optimally to the treatment [4,5], since CNV continues to develop and edema persists.

The pathogenesis of AMD is multifactorial, but exact mechanisms leading to CNV and to RPE/choroid complex degeneration are incompletely understood. Identification and modulation of other molecular targets apart from VEGF is mandatory to improve the visual prognosis of patients with wet AMD.

Glucocorticoids (GCs) are amongst the most used drugs for macular edema of many origins, including diabetic retinopathy, vein occlusion, and inflammation [6]. However, in wet AMD, intraocular GCs have been inefficient to reduce CNV and the associated macular edema [5,7], suggesting that corticoid-induced regulatory mechanisms are altered in AMD. GCs bind to the glucocorticoid (GR) and the mineralocorticoid receptor (MR), both expressed in various retinal and choroidal cells including vascular endothelial cells and retinal pigment epithelium (RPE) [8,9]. In the retina and choroid, GR pathway activation is anti-inflammatory, anti-edematous [6], but MR pathway overactivation causes inflammation, oxidative stress, and choroidal pathology [8,10,11,12]. Using transgenic approaches, we previously showed that MR invalidation in vascular endothelial cells prevented laser-induced CNV and that pharmacologic MR antagonists exerted anti-inflammatory effects and anti-angiogenic effects [13]. In a small cohort of wet AMD patients, resistant to intensive anti-VEGF treatments, we showed that the oral MR antagonist (MRA) spironolactone potentialized the anti-edematous effects of anti-VEGF and that this effect was lost after spironolactone was stopped [13]. In the search for downstream MR antagonists molecular targets, we identified that MRA anti-angiogenic effects were mediated at least in part through the up-regulation of decorin (DCN) in the RPE/choroid complex [13]. In the rat retina transcriptome, meteorin was identified also as a MR molecular target [13].

Meteorin (METRN) is a newly identified secreted protein and a member of poorly characterized, evolutionarily conserved, two-members growth factor family. The other member of the family is meteorin-like or cometin. The receptor of MTRN has not yet been characterized. During brain development, MTRN is expressed in neural stem cells and radial glial cells and in immature neurons, where it induces axonal extension [14,15]. In the retina, it promotes glial Müller cells differentiation via activation of the Jak-STAT3 pathway [15]. The potential of MTRN as a neurotrophic factor was shown by the over-expression of MTRN in brain excitotoxic models. Lentiviral MTRN overexpression in the striatum following excitotoxic injury did not enhance neurogenesis but significantly increased the proportion of new cells with astroglial and oligodendroglial features [16]. Using a cell-based encapsulated technology, the in vivo slow delivery of MTRN, protected striatal neurons from quinolinic acid-induced excitotoxicity, reduced lesion size, and improved neuronal performance [17]. More recently, MTRN was shown to reduce hyperalgesia in a chronic nerve constriction injury rat model [18]. In photochemically-induced sciatic nerve injury and chronic constriction injury of the sciatic nerve in rats, MTRN reduced signs of pain and mechanical and thermal hypersensitivity, and these effects lasted after MTRN administration, suggesting a nerve effect rather than a simple pain relief mechanism [19].

MTRN also intervenes in the glio vascular crosstalk, regulating normal angiogenesis in the brain and in the neural retina [20]. Indeed, in vitro experiments showed that MTRN exerted anti-angiogenic activity via astrocyte-derived thrombospondin-1/-2 expression and secretion, suggesting a role in angiogenesis attenuation and vascular maturation by astrocytes [20].

In the retina, MTRN expressed in astrocyte endfeet that surround blood vessels could contribute to vascular maturation and maintenance, but its exact localization in the human adult retina and its role in neovascular diseases have not been studied. The aim of this study was to confirm that MTRN expression is regulated by the mineralocorticoid pathway in the retina, to localize MTRN expression in the human retina, and to evaluate its effect in the rat laser-induced CNV model, a validated model for human wet AMD.

## 2. Material and Method

### 2.1. Ethics for Animal Use

All experiments were performed in accordance with the European Communities Council Directive 86/609/EEC and French national regulations and approved by local ethical committees. Animals were kept in pathogen-free conditions with food, water, and litter, and housed in a 12-h/12-h light/dark cycle. Anesthesia was induced by intramuscular ketamine 40 mg/kg and xylazine 4 mg/kg in rats and with topical oxybuprocain. Animals were sacrificed by carbon dioxide inhalation. Intravitreous injections (IVT) were performed using microfine (300 μL) syringes with 31G needles under topical anesthesia (tetracaine 1%, Aldrich, Lyon, France).

### 2.2. Regulation of MTRN Expression in Rat Retina by Aldosterone and/or Spironolactone, Which Regulate the Mineralocorticoid Pathway

Eight-week-old male Lewis rats were used in this experiment (ethical approval: #4488 Charles Darwin). Rat eyes received one single injection of 5 µL aldosterone 1 µM diluted in 0.9% saline, corresponding to a final concentration of 100 nM in the vitreous or 5 µL spironolactone corresponding to a final concentration of 10 µM in the vitreous. Control rat eyes were injected with 5 µL saline. Both eyes of 6 rats per group were injected.

At 24 h, rats were sacrificed by CO_2_ inhalation and eyes were enucleated and dissected on ice to retrieve the neural retina and the RPE/choroid complex. Samples were snap-frozen in liquid nitrogen and stored at −80 °C until use. Total RNA was isolated from tissues using the RNeasy Mini Kit (Qiagen, Hilden, Germany) including DNase I treatment. First-strand complementary DNA was synthesized from the total mRNA using random primers (ThermoFisher Scientific, Saint Aubin, France) and SuperScript II reverse transcriptase (ThermoFisher Scientific). Transcript levels of MTRN were analyzed by quantitative PCR performed in CFX384 Touch Real-Time PCR Detection System with SYBR Green detection using the following primers. *Mtrn*: Forward: 5′ GTG ACT TTG TGA TCC ATG GG 3′, Reverse: 5′ TGG AAC AGT GGC AGT GTC TG 3′ and *Gapdh* Forward: 5′ GAC ATG CCG CCT GGA GAA AC 3′, Reverse: 5′ AGC CCA GGA TGC CCT TTA GT 3′. Delta CT threshold calculation was used for mRNA relative quantification results.

Statistical analysis was performed using the Kruskal–Wallis non-parametric test followed by a Dunn’s multiple comparison test. *p* < 0.05 was considered significant.

### 2.3. Immunohistochemistry on Rat Retina

To localize the expression of MTRN on flat-mounted neural retina and RPE, two additional Lewis rats were used. For the retinal flat-mount, the eyes were dissected and retinas were flat-mounted, fixed 10 min in acetone at −20 °C, blocked with fetal bovine serum 10% in PBS with Triton 0.1% for 30 min, and incubated with an anti-Meteorin antibody (Abcam rabbit antibody) and with an anti-GFAP coupled to CY3 in blocking solution overnight. Retinas were then rinsed and incubated with secondary antibody (Alexa Fluor conjugated 488 anti-rabbit, Invitrogen, Carlsbad, CA, USA). Flat mounts were mounted with fluorescent aqueous mounting medium (Dako Ltd., Ely, UK). For the RPE flat-mount, following the blocking step described above, RPE flat-mounts were incubated with primary antibody anti-MTRN at dilution 1:100 (Abcam rabbit antibody) in blocking solution overnight. After rinses, flat-mounts were incubated with anti-rabbit secondary antibody at 1:200 (Molecular Probes Alexa Fluor 488), and with Rhodamin Phalloidin (Life Technologies) at a 1:300 dilution for 1 h and with DAPI at a 1:5000 dilution for 5 min under constant agitation. Flat-mounts were finally mounted with fluorescent aqueous mounting medium (Dako Ltd., UK). Images were acquired with a confocal microscope (LSM 510 Carl Zeiss, Oberkochen, Germany).

### 2.4. Immunohistochemistry on Non-Human Primate and Human Samples

Two eyes from one eight-year-old *Sapio anubis* non-human primate were used. Two hours after enucleation, the eyes were sectioned and dissected in order to retrieve the neural retina, that was fixed in 4% paraformaldehyde for 24 h at 4 °C and then rinsed in PBS.

Immunohistochemistry for MTRN, thrombospondin, and glutamine synthetase was performed in order to evaluate the distribution of MTRN in relation to glial Müller cells in the macula and fovea.

Human eyes were obtained from the oncology department of the University of Lausanne. The use of human subjects adhered to the tenets of the Declaration of Helsinki and was approved by the local Ethics Committee of the Swiss Department of Health on research involving human subjects (CER-VD 340/15 and CER-VD 19/15) and patients signed an informed consent. Two retinas were obtained from patients with anterior uveal tumors but intact retinas. One eye from a 30-year-old woman was enucleated for an untreated ciliary body melanoma. One eye from a 54-year-old woman was enucleated due to a massive peripheral nasal melanoma. In both eyes, the macula was normal. The enucleated eyes were sectioned and the anterior part (including retina up to the equator) was used for classical pathologic examination. Posterior retinas of the eyes were used for immunohistochemistry on cryosections. Due to the enucleation procedure, fresh tissues were available for analysis.

Human samples were fixed in 4% paraformaldehyde for 24 h at 4 °C. They were then rinsed in PBS 1X and included in the optimal cutting temperature for cryosectioning. For fluorescence immunohistochemistry, 10 µm thick neuroretina sections were incubated overnight in primary antibodies at 4 °C listed in (Table 1). After washing with PBS supplemented with 10% fetal calf serum and 0.1% Triton X-100, sections were incubated 3 h with their corresponding secondary antibodies AlexaFluo^®^488 or AlexaFluo^®^594 or AlexaFluo^®^647 (Table 1). All antibodies were diluted in PBS supplemented with 10% fetal calf serum and 0.1% Triton X-100. Sections were counterstained with 4′, 6-diamino-2-phenylindol (DAPI). Images were observed and captured with a confocal microscope (LSM 710 software, ZEISS, Oberkochen, Germany).

### 2.5. Laser-Induced CNV Rat Model

Eight-week-old male Long Evans rats from the Janvier Breeding Center (Le Genest-Saint-Isle, France) were used for CNV induction (local ethical committees’ approval #2541-2015110210279792 v3). After anesthesia and dilation of the pupils, coverslips were positioned on the cornea as a contact glass. Six laser burns were performed 2 to 3 optic disc diameters away from the optic nerve on both eyes with an Argon laser (532 nm) mounted on a slit lamp (175 mW, 0.1 s and 50 µm), and the rupture of Bruch’s membrane was assessed by the presence of a bubble.

### 2.6. Treatments

Treatment with MTRN was evaluated in the rat model of CNV. We used recombinant mouse MTRN protein (R&D Systems, Lille, France). After laser photocoagulation, two separate experiments were performed. In the first experiment, to test the preventive effect of MTRN, rats were injected with MTRN in the vitreous just after laser lesions were performed. They were divided into 4 treatment groups: (1) Intravitreous injection of PBS (5 µL), (2) intravitreous injection (IVT of 5 µL) of mouse recombinant MTRN (R&D System, Lille, France) at 50 g/mL in PBS, (3) IVT of 5 µL of MTRN at 200 ng/mL, (4) IVT of 5 µL of MTRN at 1 µg/mL. In a second experiment, to test the curative effect of MTRN, rats received the IVT of MTRN immediately or at seven days after laser burns were performed. They were divided into 4 groups: (1) IVT of PBS (5 µL), (2) IVT of 5 µL of MTRN at 1 µg/mL at day 0, (3) IVT of 5 µL of MTRN at 6 µg/mL at day 0, (4) IVT of 5 µL of MTRN at day 7. In both experiments, 12 eyes per group were used. Finally, to decipher the transcriptional regulations of MTRN in the neural retina and the RPE/choroid complex, we performed a third experiment using IVT of MTRN at 1 µg/mL at the time of laser burn induction and sacrificed the animals at day 7. For this experiment, 4 rats per group were used.

### 2.7. Fluorescein Angiography (FA)

FA was performed 14 days after laser induction. After pupil dilatation, fluorescein (0.2 mL of 10% fluorescein in saline) was injected intravenously in the tail of rats. Early- and late-phase angiograms were recorded 1–3 and 5–7 min, respectively, after fluorescein injection. Simultaneously, infrared images were acquired to detect the site and effective presence of laser burn. For each laser-induced lesion, fluorescein leakage was graded qualitatively by evaluating the increase in size/intensity of dye between the early and late phases. Angiographic scores were established by 2 blinded observers according to the following criteria: grade 0, no hyperfluorescence; grade 1, slight hyperfluorescence with no increase in intensity nor in size; grade 2, hyperfluorescence increasing in intensity but not in size; grade 3, hyperfluorescence increasing both in intensity and size. Mean severity gradings were compared using the Kruskal–Wallis non-parametric test followed by a Dunn’s multiple comparison test. *p* < 0.05 was considered significant. Results were also presented as the frequency of distribution of the severity grading scores as previously performed [13].

### 2.8. RPE/Choroid Flat-Mounts and CNV Quantifications

Two days after FA examination (time necessary for fluorescein elimination), eyes were enucleated, fixed in 4% PFA for 15 min at room temperature, and sectioned at the limbus; the cornea and lens were discarded. The retina was separated from the RPE/choroid complex. Eight radial incisions were made on the RPE/choroid, which was then flat-mounted and post-fixed with acetone for 15 min at −20 °C. After washing with 0.1% Triton × 100 in PBS, FITC-GSL I-Isolectin B4 (1:200, Vector, AbCys, Paris, France) was applied on two days at −4 °C. After washing with PBS, the RPE/choroid was flat-mounted and observed with a confocal microscope (Zeiss LSM710, Le Pecq, France). Images of the CNV were captured with a digital video camera coupled to a computer system. Horizontal optical sections (at 1 µm intervals) were obtained from the CNV surface. The deepest focal plane in which the surrounding choroidal vascular network connecting to the lesion could be identified was judged to be the floor of the CNV lesion. The area of CNV-related fluorescence on each horizontal section was measured using the ImageJ software. The summation of the entire fluorescent area on z-stack images from the top to the bottom of the CNV was used as an index for the CNV volume. On the same flat-mounts, MTRN immunohistochemistry was performed. Volume reduction was measured as compared to the vehicle-treated group and expressed as a percentage of reduction.

### 2.9. Statistics

Comparison between 2 groups was performed using Mann–Whitney U test. Comparison between multiple groups was analyzed using non-parametric Kruskal–Wallis followed by Dunn’s test (GraphPad Prism 5 for Windows, GraphPad Software Inc., San Diego, CA, USA). *p*-values of 0.05 or less were considered significant.

### 2.10. RNA-Sequencing Data Analysis

RNA samples from either the neural retina or the RPE/choroid complex were sent for sequencing at the iGenSeq transcriptomic platform of the Brain and Spine Institute (ICM, Paris, France). RNA quality was checked by capillary electrophoresis (Agilent 2100 Bioanalyzer system) and RNA with integrity numbers (RIN) ranging from 7.8 to 8.2 was accepted for library generation. Quality of raw data has been evaluated with FastQC. Poor quality sequences have been trimmed and adaptors removed with Fastp software to retain only good quality paired reads. Star v2.5.3a [21] has been used to align reads on reference genome rn6 using standard options. Between 30 and 38 million reads were mapped. Quantification of gene and isoform abundances has been done with rsem 1.2.28 [22], prior to normalization on library size with edgeR [23] bioconductor package. Low-expressed genes have been filtered-out. Reproducibility of replicates has been controlled with PCA representations. Finally, differential analysis has been conducted with the GLM framework likelihood ratio test from edgeR. Multiple hypothesis adjusted *p*-values were calculated with the Benjamini–Hochberg procedure to control FDR. Pheatmap Bioconductor package has been used to represent as a heatmap for the differentially expressed genes. GSEA function from clusterProfiler package has been used on HALLMARK geneset; on Gene Ontology; on Reactome gene sets; and on KEGG pathways, to get deregulated pathways from analysis, with a representation in dot plot. A genes/pathways network has been created with cytoscape, node color representing log fold change.

## 3. Results

### 3.1. MTRN Is Widely Distributed in the Rat Neural Retina and in the Retinal Pigment Epithelium and Its Expression Is Regulated by Mineralocorticoid Pathway

**Figure 1:**
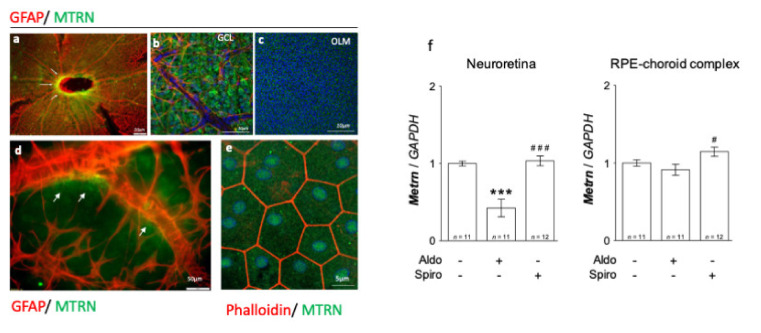
MTRN immunostaining on rat flat-mounted retina and RPE regulation in the retina and RPE/choroid complex at 24 h after MR agonist and antagonist intravitreous administration. MTRN immunostaining on the flat-mount retina (**a**–**d**). MTRN (green) is concentrated around the optic nerve head (**a**), also expressed in ganglion cell layer (GCL) (**b**), and at the outer limiting membrane (OLM). GFAP staining represents astrocytes located around vessels in the inner retina (**b**,**d**). MTRN is secreted around vessels at the endfeet of astrocytes (**d**, arrows). In RPE cells delimited by phalloidin staining, MTRN is expressed in the cytoplasm but also in the nuclei (**e**). Quantitative analysis of MTRN expression in neuroretina and RPE-choroid complex 24 hrs after aldosterone (100 nM) or spironolactone (10 µM) in vivo exposure (**f**). Significant reduction of *Mtrn* expression is observed in neural retina at 24 h after aldosterone injection (*p* < 0.001). Spironolactone up-regulated *Mtrn* compare to aldosterone, but not compared to vehicle. There is significant up-regulation of *Mtrn* expression exclusively in RPE/choroid at 24 h after spironolactone injection # (*p* < 0.05 as compared to Aldo), *** (*p* < 0.01 as compared to untreated controls), ### (*p* < 0.001, as compared to Aldo treatment).

On the neural retina flat-mount from adult rats, MTRN is concentrated around the optic nerve head (Figure 1a, arrows), all over the ganglion cell layer (GCL), and around the vessels, which are surrounded by GFAP positive astrocytes (Figure 1b). MTRN is secreted and is still visible in the deep outer nuclear layer and at the outer limiting membrane (OLM) that constitutes the junctions between the glial Müller cells and the photoreceptor segments (Figure 1c). Higher magnification shows the presence of MTRN secreted by astrocytes around the vessels (Figure 1d). On the RPE flat-mount, RPE cells are well delineated by phalloidin staining and MTRN appears as dots in the cytoplasm, but also located in the nuclei of cells (Figure 1e). Using RT-PCR, we show that *Mtrn* is expressed in the rat neural retina and in the RPE/ choroid complex and that intravitreous aldosterone injection significantly reduces *Mtrn* expression in the neural retina at 24 h (*p* < 0.001) [11]. Conversely, spironolactone up-regulates *Mtrn* expression in the RPE (*p* < 0.05) as compared to aldosterone (Figure 1f). These results show that MTRN is found not only in the neural retina but also in the RPE/choroid and is a mineralocorticoid pathway transcriptional regulated target.

### 3.2. MTRN Is Concentrated in the Macula and Co-Localized with Thrombospondin in Non-Human Primate

**Figure 2:**
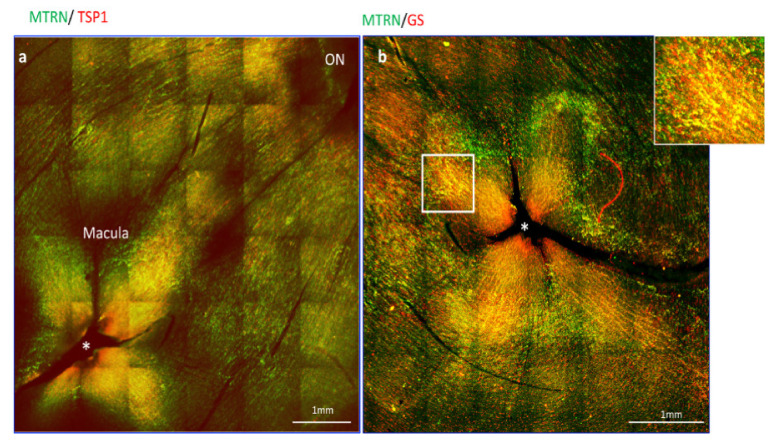
MTRN localization in non-human primate retina flat-mount. Immunostaining on the flat-mount retina of MTRN (green), TSP1 (red), and GS (red). MTRN and TSP1 are highly concentrated around the optic nerve head (NO = Optic Nerve), and co-localize at the macula and around the fovea* (**a**). Colocalization of MTRN with GS expressed in Müller cells at the macula (**b** and inset).

Since thrombospondin expression and release from astrocytes was shown to be enhanced by MRTN, we analyze the distribution of MTRN and TSP1 on flat-mounted non-human primate retina flat-mounts. Interestingly, we observed that MTRN and TSP1 are highly concentrated around the optic nerve head (Figure 2a, ON), where astrocytes density is very high, but that both proteins are also highly concentrated and co-localize at the macula and around the fovea (Figure 2a,b, star), which is avascular and devoid of astrocytes. We thus co-labelled MTRN with glutamine synthetase (GS), which is a marker of glial Müller cells, to detect potential co-localization of MTRN with these cells at the fovea. Indeed, MTRN is highly expressed in Müller glia at the fovea and in the macula, where it co-localizes with TSP1. MTRN and TSP1 could contribute to the maintenance of the avascular zone.

### 3.3. MTRN Is Distributed in All Retinal Layers of the Human Neural Retina

**Figure 3:**
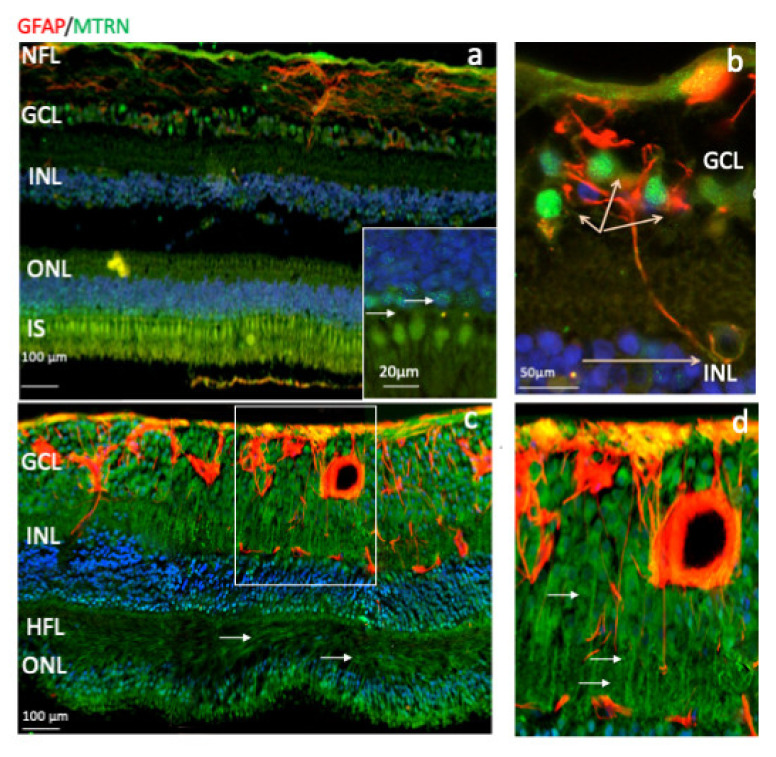
Human immunostaining of MTRN and GFAP on retinal cryosection. Immunostaining on human retinal cryosection of MTRN (green), GFAP (red), outside the macula (**a**,**b**), and in posterior retina (**c**,**d**). MTRN localized in the nerve fiber layer (NFL) (**a**), in Henle fiber layer (HFL) (**c**), in and around astrocytes, in the nuclei of ganglion cells layer (GCL), in some of the nuclei of the inner and outer nuclear layer (INL, ONL) (**a**,**c**), particularly in granules in the cone nuclei, and in the inner segments of photoreceptors (IS) (**a** and inset). MTRN is colocalized with GFAP in astrocytes and around the vessel (**b**,**d**).

**Figure 4:**
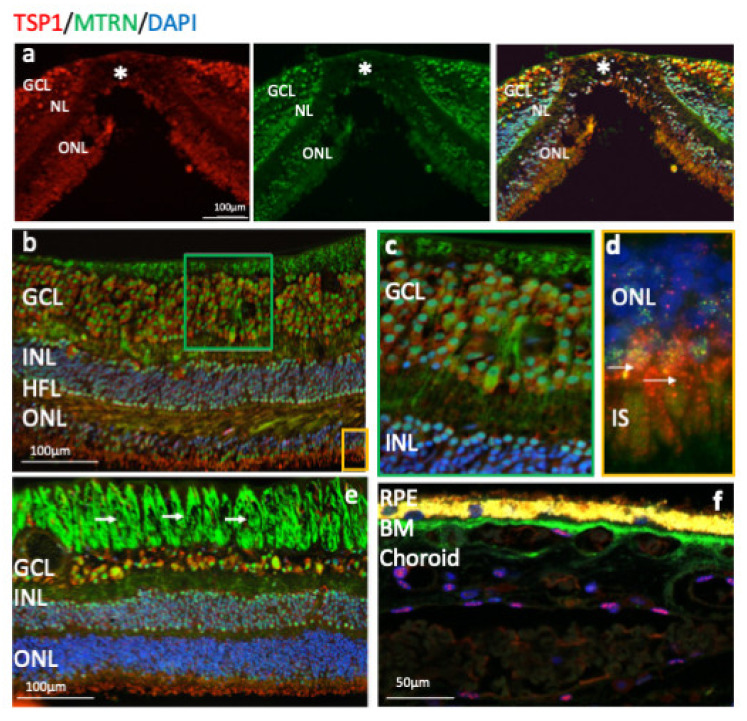
Human immunostaining of MTRN and TSP1 on retinal cryosection. Immunostaining on human retinal cryosection of MTRN (green), TSP1 (red), counter marked with DAPI (blue). MTRN (green) colocalized in all layers with TSP1, in macula (**a**) and is expressed in the cytoplasm and in nuclei of ganglion cells, in nuclei of inner and outer layers (INL, ONL) (b), in Müller fibers (**c**), and in the inner segment of photoreceptors (**d**). Close to the optic nerve, MTRN is strongly present in nerves fibers (**e**), in Bruch’s membrane (BM) (**f**). MTRN is also found as a gradient starting from the BM towards the deeper choroidal vessels (**f**).

Outside of the macula, MTRN is found in the nerve fiber layer (NFL), in and around astrocytes, in the nuclei of ganglion cells in the ganglion cell layer (GCL), in some of the nuclei of the outer nuclear layer (ONL), particularly in granules of the cone nuclei (Figure 3a, inset, arrows), and in the inner segments of photoreceptors (IS). MTRN is present in astrocytes around vessels and diffuses at the surface of the retina (Figure 3b). In the posterior retina, MTRN is localized in astrocytes, in the deepest nuclei of the inner nuclear layer (Figure 3c,d), and in the extensions of glial Müller cells of the Henle fiber layer (Figure 3c, HFL, arrows). Co-labeling of MTRN with TSP1 shows co-localization of both proteins in the macula (Figure 4a), in both cytoplasm and nuclei of ganglion cells, and mostly in nuclei of inner and outer nuclear layers (Figure 4a). In addition, secreted MTRN is observed in all retinal layers. In the macula (Figure 4b–d), MTRN co-localize with TSP1 in all layers and is present along retinal glial Müller cells (Figure 4c). In the photoreceptors, MTRN and TSP1 co-localize in cone nuclei, within nuclear bodies (Figure 4d). MTRN signal is also very strong in the nerve fibers (Figure 4e, arrows). Note that in the outer nuclear layer (which represents the nuclei of photoreceptors), MTRN expression is more intense in the macula (Figure 4b) than outside of the macula (Figure 4e). Interestingly, we observed that in human retina, MTRN is abundant in the Bruch’s membrane (BM) (Figure 4f), with a gradient towards the choroidal vessels, where TSP1 and MTRN co-localize in some nuclei of the endothelial cells.

MTRN is highly expressed in the human retina and the RPE/choroid complex and is enriched in the macula. It co-localizes with TSP1 in nuclear bodies.

### 3.4. MTRN Is Secreted and Sequestered in PML Nuclear Bodies

**Figure 5:**
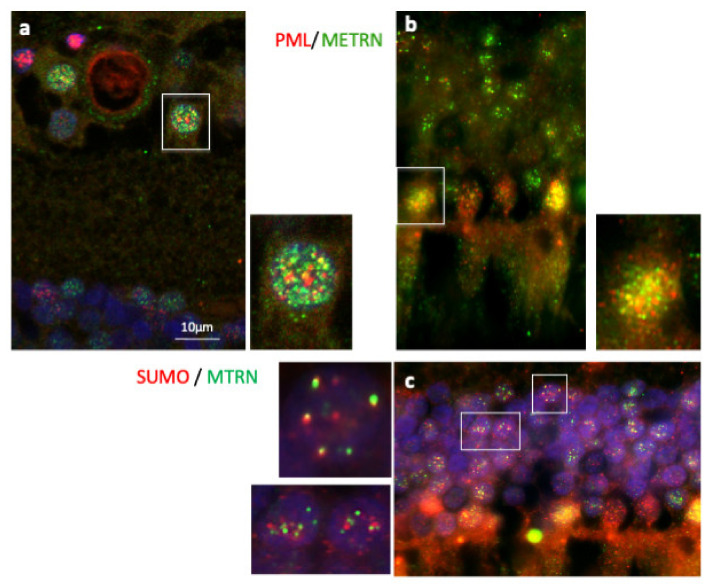
Immunostaining of PML and SUMO on human retina cryosection. Immunostaining of MTRN (green), PML (red), and SUMO (red). MTRN co-localized with PNL in nuclear ganglion cells (**a** and inset), and in nuclear photoreceptor cells (**b** and inset), but also with SUMO in cell nuclei (**c** and inset).

In order to further characterize MTRN localization in nuclear bodies, we performed a co-staining of MTRN with the promyelocytic leukemia protein (PML), known to be a key regulator of nuclear bodies organization [24] and with SUMO, as SUMOylation is an essential post-translational modification involved in partner retention in PML nuclear bodies [24]. As shown in Figure 5, both in ganglion cells (Figure 5a and inset) and in photoreceptor cells (Figure 5b and inset), MTRN co-localizes, at least in part, with PML. In addition, MTRN co-localization with SUMO (Figure 5c), suggesting that part of MTRN could be SUMOylated, which could favor its sequestration in PML nuclear bodies.

### 3.5. MTRN Reduces Laser-Induced Choroidal Neovascularization Both in a Preventive and Curative Regimen

**Figure 6:**
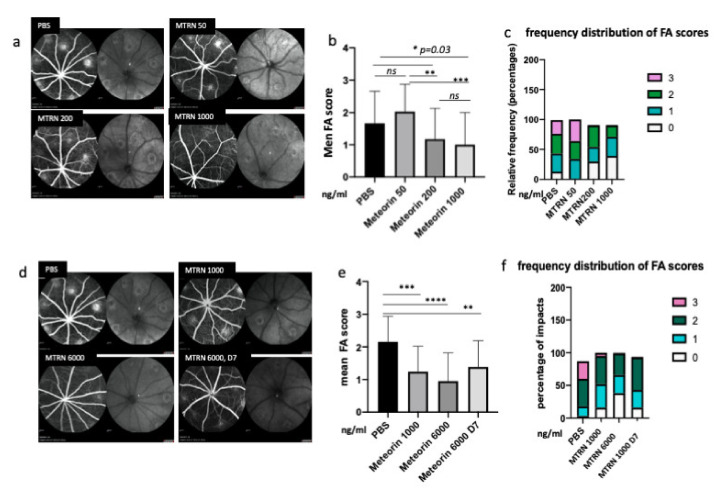
MTRN effect of CNV angiographic scores at day 14. Examples of fluorescein angiographic images at the left and infrared at the right of each eye fundus image with different tested doses (a, d). Mean grading scores and frequency of scores distribution in the preventive dose-response experiment (**b**,**c**) and in the preventive vs. curative dose experiment (**c**,**f**). MTRN: meteorin, 50 ng/mL, 200 ng/mL, 1000 ng/and 6000 mL (ng/mL), D7: treatment at day 7. Treatment. * (*p* = 0.03), ** (*p* < 0.01), *** (*p* < 0.001), **** (*p* < 0.0001).

**Figure 7:**
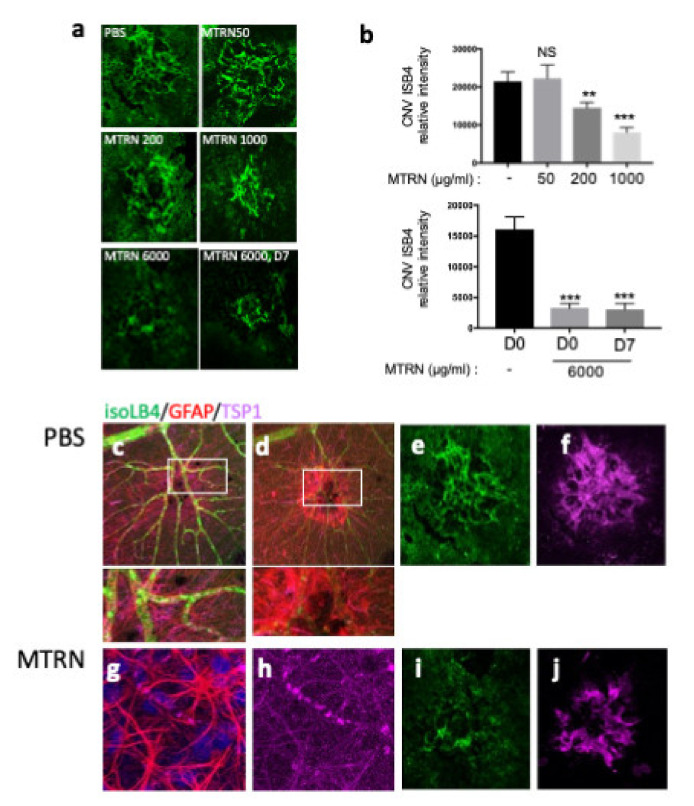
MTRN effect on CNV quantified by isolectin B4. Choroidal flat-mount laser impact images, for each treatment, stained with ß4-isolectin (green) (**a**). Quantification of neovascular volume (**b**). Immunostaining of isoLB4 (green), GFAP (red), and TSP1 (purple) on neuroretina treated by PBS (**c**,**d** and insets) and MTRN (**g**,**h** and insets), and on laser impacts of RPE-choroid complex treated by PBS (**e**,**f**) and MTRN (**i,j**). * (*p* = 0.03), ** (*p* < 0.01), *** (*p* < 0.001).

At preventive doses, MTRN reduced the angiographic leakage scores in a dose dependent manner with a significant reduction from 1 µg/mL (5 µL corresponding to a final concentration of 1 ng/µL in the vitreous), although a reduction of the 3 grading score frequency was already observed with the lower dose of 200 ng/mL (0.2 µg/mL final vitreous concentration). When administered at day 7, when CNV has already begin to form, MTRN was still efficient, although at a higher dose (6 ng/µL final vitreous concentration) (Figure 6). Reduction of CNV was further confirmed by quantification of neovascular membrane surface on flat-mounted choroids (Figure 7a), showing a 62% reduction in the preventive MTRN 1000 group and a 31% CNV reduction in the MTRN 200 group. When MTRN was administered at high dose (MTRN 6000 group), 80% of CNV reduction was observed, whether at a curative or preventive dose (Figure 7b). MTRN significantly reduced CNV when administered at the time of laser burn for the 200, 1000, and 6000 doses and when administered at the highest dose at day 7 (*p* < 0.01 for the 200 dose and <0.001 for other doses). At the level of the laser burn, on the neural retina, GFAP activation could be detected either in the superficial (Figure 7c, inset) or in the deep layers (Figure 7d inset), associated with TSP1 diffusion around the activated glial cells in eyes treated with PBS. Intense TSP1 staining was also observed at the level of CNV (Figure 7f). In the MTRN 1000 treated eye, high TSP1 signal could be observed at the endfeet of astrocytes on the vessel (Figure 7g,h) and despite the reduced size of CNV, TSP1 signal appears larger than the CNV (Figure 7i,j). Altogether, these results show that MTRN has an anti-angiogenic effect on CNV and that this effect could be mediated, at least in part, through TSP1.

### 3.6. MTRN Regulates Genes and Pathways Involved in Angiogenesis Independent from VEGF in the RPE/Choroid and in the Retina

**Figure 8:**
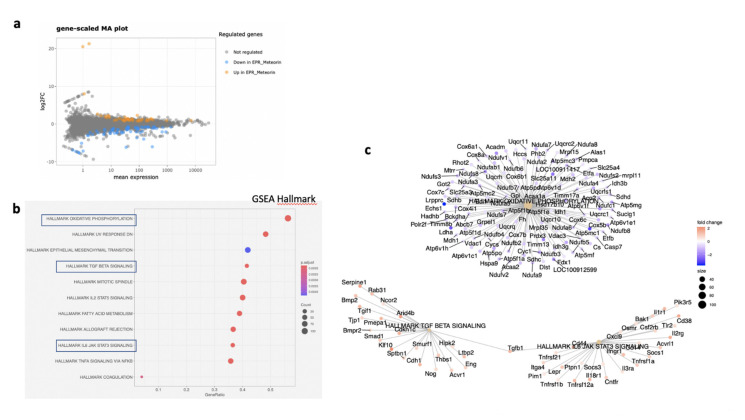
Transcriptomic analysis of the neural retina at day 7 after MTRN (1 µg/mL) was administered at the time of laser delivery. Gene-scaled MA plot. (EdgeR, log2 FC > 0.5, *p* < 0.05) (**a**), GSEA Hallmark. The dot plot depicts the gene ratios (number of core genes over the total number of genes in the set). The dots are colored by the adjusted *p*-value and their size is proportional with the size of the gene set (**b**), genes in the pathways in boxplots are detailed in the gene concept network, (**c**) gene pathway association using the Gene-Concept Network that provides information on the linkages of genes and pathways. Genes are colored by their FC and the size of the dots are proportional to the size of the gene set.

**Figure 9:**
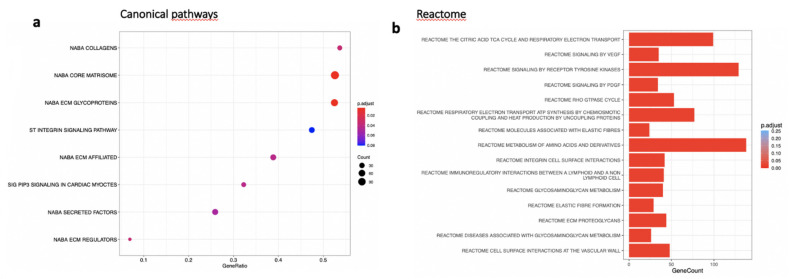
Transcriptomic analysis of the neural retina at day 7 after MTRN (1 µg/mL) was administered at the time of laser delivery. (**a**) Enriched term plot from canonical pathways gene sets from pathway databases. The dot plot depicts the gene ratios (number of core genes over the total number of genes in the set). The dots are colored by the adjusted *p*-value and their size is proportional with the size of the gene set (**b**), Reactome-enriched term plots from Reactome gene sets. The dot plot depicts the gene ratios (number of core genes over the total number of genes in the set). The dots are colored by the adjusted *p*-value and their size is proportional with the size of the gene set.

In the RPE/choroid complex at 7 days after laser induction and MTRN treatment, 42 genes were significantly up-regulated and 69 genes were significantly down-regulated (EdgeR, log2 FC > 0.5, *p* < 0.05). A full list of differentially regulated genes is available in Supplementary Table S1. The gene-scaled MA plot showed homogenous regulations of highly and poorly expressed genes (Figure 8a). GSEA, using the Hallmark gene sets, was identified as regulated in coagulation, TNFA via NFkB, IL6-Jak-STAT3 signaling, allograft rejection, fatty acid metabolism, IL2 STAT5 signaling, mitotic spindle, TGF beta signaling, epithelial mesenchymal transition, and oxidative phosphorylation (Figure 8b). In TGF beta signaling and IL6 Jak-STAT5 signaling pathways, genes encoding proteins were involved in barrier properties such as *Tjp1* and *Cdh1*, and genes encoding proteins involved in angiogenesis such as *Tgf beta*, *Il18*, and Thbs1, were up-regulated (Figure 8c). In the oxidative phosphorylation pathway, all genes were down-regulated (Figure 8c). GSEA, using the canonical pathway analysis, shows that MTRN regulates pathways in the extra cellular matrix, core matrisome, ECM glycoproteins, collagens, and secreted factors. Genes encoding decorin, biglycan, versican, aggregan, and the small leucin rich protein 2a were regulated by MTRN in these pathways. Reactome pathways analysis showed regulation of extracellular matrix, proteoglycan and glycosaminoglycan (Figure 9), VEGF and PDGF signaling, and cell surface interactions at the vascular wall. In the VEGF, Reactome pathway, genes encoding *Vegf*, or its receptors were not down-regulated (see Supplementary Table S1).

**Figure 10:**
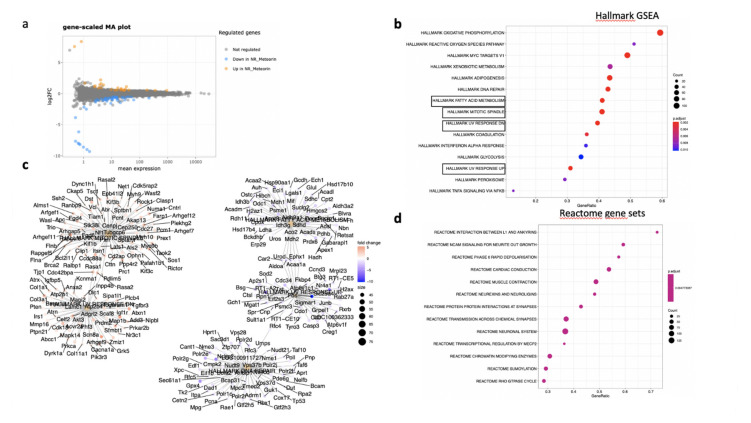
Transcriptomic analysis of the RPE-choroid complex at day 7 after MTRN (1 µg/mL) was administered at the time of laser delivery. Gene-scaled MA plot. (DESeq2, log2 FC > 1, *p* < 0.05) (**a**), GSEA Hallmark. The dot plot depicts the gene ratios (number of core genes over the total number of genes in the set). The dots are colored by the adjusted *p*-value and their size is proportional with the size of the gene set (**b**), genes in the pathways in boxplots are detailed in the gene concept network, (**c**) gene pathway association using the gene concept network that provides information on the linkages of genes and pathways. Genes are colored by their FC and the size of the dots are proportional to the size of the gene set. (**d**) Reactome-enriched term plots from Reactome gene sets. The dot plot depicts the gene ratios (number of core genes over the total number of genes in the set). The dots are colored by the adjusted *p*-value and their size is proportional with the size of the gene set.

In the neural retina, 22 genes were significantly down- and 10 genes were up-regulated (DESeq2, log2FC > 1, *p* < 0.05). A full list of regulated genes is available in Supplementary Table S2. The gene-scaled MA plot showed homogenous regulations of highly and poorly expressed genes (Figure 8a). GSEA, using the Hallmark gene sets (Figure 10b), identified TNFA signaling via NFKB, several pathways involved in oxidative stress such as peroxisome (all genes are down-regulated), reactive oxygen species (all genes down-regulated), UV-response down (all genes up), UV-response up (all genes down-regulated), DNA repair (all genes down-regulated) (Figure 10c), indicating reduction of oxidative stress markers in MTRN-treated retina. Additional regulated pathways were coagulation (all genes down-regulated including *Mmp3, Timp1, Clu, Sparc, S100a1, Thbd*, and others), oxidative phosphorylation (all down-regulated), and mitotic spindle (all up-regulated including *Cdc42, Rock1*, and *2*). These regulated pathways indicate that MTRN reduces oxidative stress, activates microtubules formation and organization, and acts on fatty acid and mitochondria metabolism.

Reactome pathway analysis (Figure 10d) identified SUMOylation and several pathways involved in the neural system, neurite outgrowth, synapses, neuroligin, and neurexin, suggesting an effect on neural structural organization.

## 4. Discussion

In the first part of this study, we confirmed that MTRN is a mineralocorticoid target gene. Indeed, in our previous study, we showed that antagonism of the mineralocorticoid receptor (MR) pathway had anti-angiogenic effect in the laser-induced CNV model in rodents and that this effect was unrelated to VEGF, but rather mediated by decorin [13] and by other proteins, up-regulated by MR, and detected in a transcriptomic study, including MTRN [11,13]. Aldosterone, a specific MR agonist, significantly down-regulated MTRN in the retina in vivo, whilst spironolactone rather increased its expression as compared to aldosterone in the RPE/choroid complex. These differences in agonist and antagonists effects could be related to the biodisponibility of spironolactone that has a very short half-life in the vitreous and strong hydrophobicity [25] and could thus be rapidly eliminated in the deep retinal layers.

Having identified MTRN as a potential anti-angiogenic effect of spironolactone, we then have studied its distribution in the adult retina. Immunolocalization of MTRN in the rodent and human retina has confirmed that in adults, MTRN is expressed and secreted by glial cells including astrocytes and glial Müller cells [20]. MTRN was shown to promote GFAP expression in astrocytes, favoring their differentiation via the Jak-STAT3 pathway [15]. Interestingly, our study shows that MTRN is enriched in the macula of non-human primates and in humans, where it is mostly expressed and secreted by glial Müller cells, which have specific features and organization in the macula [26], but it is also expressed in cone photoreceptors and localized in nuclear bodies, including PML ones. In the macula, MTRN associates with thrombospondin-1, which is produced by glial cells and is a strong negative regulator of angiogenesis [27]; it could be hypothesized that MTRN could contribute to the maintenance of the avascular foveal zone. In addition, we clearly identified MTRN in RPE cells both in rats and in humans, where it is secreted towards the Bruch’s membrane and the choroidal vessels and is also sequestered in nuclear bodies in RPE. The expression of MTRN in human RPE cells derived from iPSc was confirmed as well (manuscript under revision). However, MTRN was also found in ganglion cells and in nerve fibers and in some nuclei of the deeper inner nuclear layer. MTRN is thus ubiquitous in the human retina and choroid. The localization of MTRN in nuclear bodies was further shown by SUMOylation of the protein, which is a process that favors its retention in PML nuclear bodies [24,28]. Indeed, PML nuclear bodies can act as reservoirs for stress-response proteins [29,30], which can be very quickly released in case of stress such as hypoxia, known to be a strong enhancer of MTRN production [20]. In the neural retina, Reactome pathway analysis, SUMOylation was regulated by MTRN, confirming that this protein modification could regulate its activity.

In the laser-induced CNV model, intravitreous injection of MTRN was performed either preventively at increasing doses or curatively at 7 days after laser induction. The mouse recombinant MTRN has 87% of sequence homology with the rat and a molecular weight of 30KD, which is comparable to therapeutic proteins injected in humans to treat wet AMD with monthly injections [31], suggesting that a single injection should cover the duration of the experiment, which was 15 days. However, in order to reach efficient concentrations of therapeutic proteins in the deep retinal layers, and maintain these levels for several weeks, high initial concentrations are required. This is confirmed in our experiment, since an estimated initial concentration of 1 to 6 ng/mL was required to reduce significantly the angiographic leakage and size of the choroidal neovascular membrane. The highest MTRN dose tested (6 ng/mL in the vitreous) maintained an efficient anti-angiogenic effect both on leakage and CNV size when injected at 7 days, which is relevant in the prospect of clinical use of MTRN. We can ensure that MTRN activity was maintained for at least 7 days since transcriptional regulations in the neural retina and in the RPE/choroid complex could be observed at this time point.

The mechanisms of action of MTRN on CNV are complex and not related to VEGF expression regulation, since we did not observe any decrease in VEGF or its receptors in the transcriptomic study and using RT-PCR at day 3 and 7 (not shown). The transcriptomic analysis at seven days did not show a significant down-regulation of VEGF or its receptors nor an increased expression of thrombospondin-1, although GSEA Hallmark pathway analysis revealed an increase in *Thsp1* and *Thsp2* gene expression in RPE/choroid-treated rats. Accordingly, we observed strong thrombospondin release from astrocytes endfeet at the site of laser burn at 14 days and high thrombospondin expression in the CNV lesion despite its significantly reduced size. It can thus be hypothesized that MTRN act, at least in part, through secretion of thrombospondin, known to be reduced in eyes with wet AMD [32].

The transcriptomic analysis provides a broader view of the potential transcriptional mechanisms of action of MTRN. Although it gives a snapshot picture of expression levels at a single time point after treatment (day 7), it indicates major pathways regulated in RPE/choroid and in the retina treated with MTRN. As expected, the IL6-Jak-STAT3 pathway is regulated by MTRN, as already demonstrated in the brain and the retina [15,33].

Interestingly, MTRN regulates in RPE/choroid numbers of genes encoding proteins and glycoproteins involved in Bruch’s membrane maintenance including decorin, biglycan, mimecan, and aggregan, amongst others, and is known to be de-regulated in AMD [34]. Decorin gene, up-regulated by MTRN, was shown to decrease hypoxia-induced Met, Rac1, HIF-1α, and VEGF expression in ARPE-19 cells in vitro [35], but to exert anti-angiogenic activity in the CNV model, without reducing VEGF [13]. MTRN also regulates metalloprotease expression and particularly down-regulates TIMP1, MMP10, and MMP3, involved in AMD. MMP3, mostly located in the Bruch membrane [36], is up-regulated in RPE, submitted to oxidative stress [37], and in complement activation-induced injury [38]. In the TGF beta signaling pathway, the gene encoding thrombospodin-1 was up-regulated, indicating potential additional anti-angiogenic effect through thrombospondin-1 regulation [27]. Altogether, these results indicate that MTRN plays an important role in Bruch’s membrane/RPE complex integrity and could regulate choroidal neovascularization through extracellular matrix and endothelial cells interactions.

In the neural retina, MTRN regulates several pathways that represent oxydative stress, mostly towards a reduction of oxydative stress, involving matrisome and mitochondria. Moreover, Reactome pathways show that MTRN interact with neurite outgrowth, synapses and the tubulin-associated proteins, which is in line with its effects on nerves both in pain and in excitotoxic injury models [16,17,19,33]. Whether MTRN could play additional neuroprotective and antifibrotic effects in AMD remains to be demonstrated.

Like in any study, there are limitations due to experimental potential biais. Here, we analyzed the localization of MTRN on human retina from individuals that have been enucletaed, which offers the unique opportunity to analyze fresh tissue. On the other hand, these eyes have anterior tumors that have indicated the enucleation. Thus, although the posterior retina did not show morphological changes and GFAP, which is a very sensitive stress marker in retinal Müller cells, was not activated, we cannot exclude potential pathological influence on the results. On the other hand, MTRN enrichment in the macula was not only observed in humans, but also in non-human primate macula, and we also found similar enrichment using differential transcriptomic analysis of macula versus periphery in monkeys (personnal unpublished data). Nevertheless, these results should be confirmed in non-pathologic human eyes from donors of different ages.

In conclusion, we report herein that MTRN is a ubiquitous protein that is not only secreted by glial cells in the human retina but also sequestered in nuclear bodies in photoreceptor and ganglion cells. It is a target gene of the MR antagonist, that through its anti-angiogenic effects, it could contribute to the effect observed in patients with wet AMD treated with spironolactone in addition to anti-VEGF [13].

Further studies should validate the regulatory target genes, identified by a single time point transcriptomic analysis, and evaluate potential synergic effects of MTRN with currently used anti-VEGF drugs.

## Figures and Tables

**Table 1:** List of antibodies.

Antibodies	Species	Reference	Lab provider	Dilution
Anti GFAP Antibody, Cy3 Conjugate	Mouse	MAB3402C3	Dako cytomation	1/200
Anti Meteorin	Rabbit	Ab12956	Abcam	1/100
Anti Glutamine synthetase clone GS-6	Mouse	MAB 302	Merck Millipore	1/300
Anti TSP1	Mouse	MA5-13398	Invitrogen	1/100
SUMO1	Mouse	Sc5308	Santa Cruz	1/100
PML	Mouse	PG-M3 sc-966	Santa Cruz	1/100
AlexaFluo® 488 – AlexaFluo®594 – AlexaFluo®647		Ab12956	Invitrogen/Thermofischer	1/300
4′, 6′ –diamino-2-phenylindo DAPI			Sigma-Aldrich	1/5000

## Data Availability

All raw data are available upon reasonable request.

## Supplementary Materials

The following are available online at https://www.mdpi.com/article/10.3390/jcm10132973/s1, Table S1: DE genes RPE/Choroid; Table S2: DE genes Retina.
