# Supplementary material for: Meteorin Is a Novel Therapeutic Target for Wet Age-Related Macular Degeneration
Source: J Clin Med. 2021 Jul 2;10(13):2973. doi: 10.3390/jcm10132973 (PMC8268911; doi:10.3390/jcm10132973)
Supplement: Supplementary file 1 [file jcm-10-02973-s001.zip › jcm-1264774-supplementary.pdf]

supplementray Table 1 DE genes RPE/Choroid

FeatureID	EntrezID	Gene Name	TranscriptType	GeneBiotype	logFC	FC	PValue	FDR	Status
AABR07028839.1	 NA 	NA	NA	NA	-1.84828071812607	-3.6007082731498	0.000130478864278073	0.0255636902142729	Down in EPR_Meteorin
AABR07029836.1	 NA 	NA	NA	NA	1.65640105502531	3.15229172848854	2.07130796254484e-05	0.00747892768863177	Up in EPR_Meteorin
AABR07029907.2	 NA 	NA	NA	NA	-7.73301022714488	-212.749248724318	4.45536887874845e-05	0.0126818292273206	Down in EPR_Meteorin
AABR07044711.1	 NA 	NA	NA	NA	-0.840892576196551	-1.79115796468691	7.44431337160214e-06	0.00330308563305852	Down in EPR_Meteorin
AABR07052523.1	 NA 	NA	NA	NA	-1.24115698871656	-2.36388030855026	2.74158309074309e-06	0.00169674563960406	Down in EPR_Meteorin
AABR07057438.1	 NA 	NA	NA	NA	-5.12904759242854	-34.9942890315887	0.000197034757226984	0.0333984982868121	Down in EPR_Meteorin
AABR07063248.1	 NA 	NA	NA	NA	2.61933849119444	6.1446826010382	2.5626044312791e-05	0.00859098898895033	Up in EPR_Meteorin
Abcg2	 312382 	ATP binding cassette subfamily G member 2	protein_coding	protein_coding	2.04479219986669	4.12613836162135	0.000254293638536338	0.0387502407167595	Up in EPR_Meteorin
AC108574.1	 NA 	NA	NA	NA	1.01585852096215	2.02210585255868	6.95113699859846e-06	0.00317772281093504	Up in EPR_Meteorin
Acer2	 313339 	alkaline ceramidase 2	protein_coding	protein_coding	1.30495672900371	2.47076316712509	9.19392647432606e-06	0.00374863715653197	Up in EPR_Meteorin
Adgrl4	 64124 	adhesion G protein-coupled receptor L4	NA	NA	1.17621971042658	2.25983855750705	0.00023628741023234	0.036748782172836	Up in EPR_Meteorin
Aipl1	 59110 	aryl hydrocarbon receptor-interacting protein-like 1	protein_coding	protein_coding	-2.02293351432592	-4.06409327653152	0.000163008314082202	0.0294878988865758	Down in EPR_Meteorin
Atp6v0e2	 436582 	ATPase, H+ transporting V0 subunit e2	protein_coding	protein_coding	-1.03791573918584	-2.05325916703699	3.20593395455526e-05	0.0100759832580043	Down in EPR_Meteorin
Atp6v1b2	 117596 	ATPase H+ transporting V1 subunit B2	protein_coding	protein_coding	-0.889492004104625	-1.85252370583026	1.52734103131239e-05	0.00576036669959468	Down in EPR_Meteorin
Calml4	 691455 	calmodulin-like 4	protein_coding	protein_coding	-1.35639823226388	-2.56045150072229	9.73563587961419e-05	0.0211082284822366	Down in EPR_Meteorin
Ccnj	 294053 	cyclin J	protein_coding	protein_coding	8.0107748549131	257.919109007937	2.13173731016284e-05	0.00747892768863177	Up in EPR_Meteorin
Cdh11	 84407 	cadherin 11	protein_coding	protein_coding	0.944959356407595	1.92513465056256	1.68082967933217e-06	0.00126784982712026	Up in EPR_Meteorin
Cers1	 290658 	ceramide synthase 1	protein_coding	protein_coding	21.3059845782098	2592629.42174806	1.34715387293836e-06	0.00114104896122474	Up in EPR_Meteorin
Ckmt1	 29593 	creatine kinase, mitochondrial 1	NA	NA	-1.57510540983866	-2.97957261937287	0.000224282928713848	0.0363820673395388	Down in EPR_Meteorin
Clta	 83800 	clathrin, light chain A	protein_coding	protein_coding	-0.750263287832745	-1.68209978102979	0.000336421363778074	0.0474322681678133	Down in EPR_Meteorin
Crx	 60446 	cone-rod homeobox	protein_coding	protein_coding	-2.55136453137402	-5.86188445651031	2.76152385292715e-05	0.0090565975750563	Down in EPR_Meteorin
Ctnna2	 297357 	catenin alpha 2	protein_coding	protein_coding	-2.36400170461928	-5.1479630717878	3.46490415265407e-05	0.0106676620503958	Down in EPR_Meteorin
Dapl1	 362136 	death associated protein-like 1	protein_coding	protein_coding	-2.3011567022367	-4.92852759010921	0.000152620175479814	0.0281522917770906	Down in EPR_Meteorin
Dnase2b	 59296 	deoxyribonuclease 2 beta	protein_coding	protein_coding	-3.06327333940477	-8.3586696235393	6.40755092956766e-05	0.0160411502527415	Down in EPR_Meteorin
Dusp7	 300980 	dual specificity phosphatase 7	protein_coding	protein_coding	0.840859769870012	1.79111723491152	4.5617892278807e-06	0.00237307421695891	Up in EPR_Meteorin
Ebf1	 116543 	EBF transcription factor 1	protein_coding	protein_coding	0.887394365373747	1.84983214474515	9.79435233830413e-05	0.0211082284822366	Up in EPR_Meteorin
Echs1	 140547 	enoyl-CoA hydratase, short chain 1	protein_coding	protein_coding	-1.1392506460024	-2.20266584215823	0.000182652190223034	0.031312397064826	Down in EPR_Meteorin
Edn3	 366270 	endothelin 3	protein_coding	protein_coding	0.973077440438081	1.96302349531081	6.1371008071612e-05	0.0156922547079379	Up in EPR_Meteorin
Eno1	 24333 	enolase 1	protein_coding	protein_coding	-0.925222325080092	-1.89897685928825	2.16296043867582e-06	0.00148320096263016	Down in EPR_Meteorin
Eno2	 24334 	enolase 2	protein_coding	protein_coding	-1.27641296915051	-2.42235946519555	4.61459113694832e-08	5.35505553015403e-05	Down in EPR_Meteorin
Erg	 170909 	ETS transcription factor ERG	protein_coding	protein_coding	1.17188595948084	2.25306035259935	3.71176147959358e-06	0.00199984406004103	Up in EPR_Meteorin
Esrrb	 299210 	estrogen-related receptor beta	protein_coding	protein_coding	-2.84108913036541	-7.16560804162378	4.78377753936877e-06	0.00240560226529724	Down in EPR_Meteorin
F13a1	 60327 	coagulation factor XIII A1 chain	protein_coding	protein_coding	-1.55943111483241	-2.9473759927187	5.97238649533543e-05	0.0156922547079379	Down in EPR_Meteorin
F2r	 25439 	coagulation factor II (thrombin) receptor	protein_coding	protein_coding	1.40552838085646	2.64914788216249	6.00007624363368e-06	0.00282866094410805	Up in EPR_Meteorin
Fam169a	 310013 	family with sequence similarity 169, member A	protein_coding	protein_coding	-1.53954414032281	-2.90702633357297	0.000167848517514838	0.0297139834999513	Down in EPR_Meteorin
Fam89a	 361441 	family with sequence similarity 89, member A	protein_coding	protein_coding	-2.38794341198252	-5.2341069800986	0.000112475281577303	0.0235666958038221	Down in EPR_Meteorin
Fdft1	 29580 	farnesyl diphosphate farnesyl transferase 1	protein_coding	protein_coding	-0.730878890499253	-1.65964984318959	0.000286321261431012	0.0423474759798848	Down in EPR_Meteorin
Fgd5	 362402 	FYVE, RhoGEF and PH domain containing 5	protein_coding	protein_coding	0.99585653137352	1.99426417349355	0.000112074368116607	0.0235666958038221	Up in EPR_Meteorin
Fkbp5	 361810 	FKBP prolyl isomerase 5	protein_coding	protein_coding	1.8280661107068	3.55060805001127	3.62982379697933e-11	9.12658696687169e-08	Up in EPR_Meteorin
Fmo2	 246245 	flavin containing dimethylaniline monoxygenase 2	protein_coding	protein_coding	2.17856744895821	4.52703810143509	8.65693266014895e-06	0.00362773572530575	Up in EPR_Meteorin
G6pd	 24377 	glucose-6-phosphate dehydrogenase	protein_coding	protein_coding	-0.849466785360072	-1.80183485078228	0.000262056872731088	0.039533899820212	Down in EPR_Meteorin
Ggt6	 408206 	gamma-glutamyl transferase 6	protein_coding	protein_coding	-2.29706379665468	-4.91456524136888	0.00021484022624262	0.0358784801093923	Down in EPR_Meteorin
Gip	 25040 	gastric inhibitory polypeptide	protein_coding	protein_coding	-2.30193358115726	-4.93118227469418	1.05292817863819e-08	1.58844745029357e-05	Down in EPR_Meteorin
Gnat1	 363143 	G protein subunit alpha transducin 1	protein_coding	protein_coding	-2.10763649475426	-4.30984652306595	0.000119994574897357	0.0243440023844834	Down in EPR_Meteorin
Grk1	 81760 	G protein-coupled receptor kinase 1	protein_coding	protein_coding	-2.36059364940248	-5.13581647419147	0.000265373968393508	0.0396379374968759	Down in EPR_Meteorin
Gstp1	 24426 	glutathione S-transferase pi 1	protein_coding	protein_coding	-1.19174900989369	-2.28429505326971	2.92426001787787e-06	0.00169674563960406	Down in EPR_Meteorin
Hes1	 29577 	hes family bHLH transcription factor 1	protein_coding	protein_coding	0.972181371902703	1.96180462549665	0.000120782228574054	0.0243440023844834	Up in EPR_Meteorin
Hpgd	 79242 	15-hydroxyprostaglandin dehydrogenase	protein_coding	protein_coding	-4.01986838567233	-16.2218717185969	1.82149226393257e-42	9.15967743122891e-39	Down in EPR_Meteorin
Hspa1b	 24472 	heat shock protein family A (Hsp70) member 1B	protein_coding	protein_coding	0.969773765279542	1.95853344570909	0.00025415307211189	0.0387502407167595	Up in EPR_Meteorin
Igfbp3	 24484 	insulin-like growth factor binding protein 3	protein_coding	protein_coding	1.23692319399854	2.35695333312368	6.59254484734172e-05	0.0160411502527415	Up in EPR_Meteorin
Kcne2	 171138 	potassium voltage-gated channel subfamily E regu	protein_coding	protein_coding	-1.74920983598242	-3.36174392968374	2.47557686287874e-05	0.00848785285304288	Down in EPR_Meteorin
Kcnj14	 276720 	potassium inwardly-rectifying channel, subfamily J	protein_coding	protein_coding	-2.42906878169535	-5.38545702716459	2.11449002275853e-05	0.00747892768863177	Down in EPR_Meteorin
Klf9	 117560 	Kruppel-like factor 9	protein_coding	protein_coding	1.2017469739625	2.30018033584035	3.67915535594383e-05	0.0111007475399537	Up in EPR_Meteorin
Lamp3	 303801 	lysosomal-associated membrane protein 3	protein_coding	protein_coding	-3.06307978406616	-8.35754828010905	0.000153021869662033	0.0281522917770906	Down in EPR_Meteorin
Ldha	 24533 	lactate dehydrogenase A	protein_coding	protein_coding	-1.16656738017745	-2.24476960592163	1.25126631226976e-05	0.00496752725971093	Down in EPR_Meteorin
LOC100364062	 100364062 	M2 pyruvate kinase-like	NA	NA	-1.18276359247025	-2.27011218139668	7.66590689356327e-06	0.00330422489703701	Down in EPR_Meteorin
LOC100911725	 100911725 	6-phosphofructo-2-kinase/fructose-2,6-bisphosph	NA	NA	-6.93934597895613	-122.730157049583	0.000218800223390169	0.0358784801093923	Down in EPR_Meteorin
LOC100912399	 100912399 	mitogen-activated protein kinase kinase kinase 1-li	protein_coding	protein_coding	20.5288255666443	1512837.46556109	3.23482096358917e-06	0.00180742626135949	Up in EPR_Meteorin
LOC102556337	 102556337 	mitochondrial fission factor-like	protein_coding	protein_coding	-2.5310523920908	-5.77993148858034	1.93197555528334e-08	2.64961665700041e-05	Down in EPR_Meteorin
LOC108348055	 108348055 	B9 domain-containing protein 1	NA	NA	-7.57665598797103	-190.897710245174	6.4946212519021e-05	0.0160411502527415	Down in EPR_Meteorin
LOC108351137	 108351137 	glyceraldehyde-3-phosphate dehydrogenase	NA	NA	-0.809903304211234	-1.7530939386015	0.000309364497169554	0.0448757000413451	Down in EPR_Meteorin
LOC685431	 685431 	similar to Alpha-enolase (2-phospho-D-glycerate h	pseudogene	pseudogene	-1.08381759634975	-2.11963755861594	2.46808444745351e-06	0.00161884878149059	Down in EPR_Meteorin
Map3k6	 313022 	mitogen-activated protein kinase kinase kinase 6	protein_coding	protein_coding	2.03241460953591	4.09088961559652	4.00867418955641e-08	5.03957156863733e-05	Up in EPR_Meteorin
Marcks	 25603 	myristoylated alanine rich protein kinase C substra	protein_coding	protein_coding	0.787194862777564	1.72571576333049	0.000164190872760995	0.0294878988865758	Up in EPR_Meteorin
Mfap4	 287382 	microfibril associated protein 4	NA	NA	1.32589641554162	2.50688604181855	1.50427359318036e-05	0.00576036669959468	Up in EPR_Meteorin
Mfge8	 25277 	milk fat globule EGF and factor V/VIII domain cont	protein_coding	protein_coding	-0.771265344001851	-1.70676607995159	0.000300413830210085	0.0440004178888286	Down in EPR_Meteorin
Mmp10	 117061 	matrix metallopeptidase 10	protein_coding	protein_coding	-6.20105825027051	-73.5706407032332	6.26322061747057e-26	2.36217365587903e-22	Down in EPR_Meteorin
Mmp3	 171045 	matrix metallopeptidase 3	protein_coding	protein_coding	-6.05965593214801	-66.7018990834327	6.29647162658775e-64	9.49885709587028e-60	Down in EPR_Meteorin
Mpp4	 58808 	membrane palmitoylated protein 4	protein_coding	protein_coding	-2.21015160049223	-4.62723894707025	6.13629081079108e-05	0.0156922547079379	Down in EPR_Meteorin
Ndst2	 114002 	N-deacetylase and N-sulfotransferase 2	protein_coding	protein_coding	1.0741457641571	2.10547502747805	0.000169389008418123	0.0297139834999513	Up in EPR_Meteorin
Net1	 307098 	neuroepithelial cell transforming 1	protein_coding	protein_coding	1.04337536158892	2.06104408163972	8.9324025694669e-09	1.49726916847753e-05	Up in EPR_Meteorin
Nrl	 290221 	neural retina leucine zipper	protein_coding	protein_coding	-2.45227657492272	-5.4727902879571	7.18116005814359e-05	0.0171960286725642	Down in EPR_Meteorin
Nxnl1	 306342 	nucleoredoxin-like 1	protein_coding	protein_coding	-1.36665834664665	-2.57872574532899	5.589921091736e-05	0.0150588481410588	Down in EPR_Meteorin
Ogn	 291015 	osteoglycin	protein_coding	protein_coding	1.34134066190876	2.53386675583359	0.000360222055976283	0.0495713090064465	Up in EPR_Meteorin
Pde6a	 307401 	phosphodiesterase 6A	protein_coding	protein_coding	-2.00777316584534	-4.02160995634127	0.000226969700310367	0.036426222328534	Down in EPR_Meteorin
Pdilt	 293544 	protein disulfide isomerase-like, testis expressed	protein_coding	protein_coding	1.25418519553706	2.38532392370846	3.91889152649598e-05	0.0113693072247535	Up in EPR_Meteorin
Pgam1	 24642 	phosphoglycerate mutase 1	protein_coding	protein_coding	-0.785417381810985	-1.72359089425475	5.27978375352188e-06	0.00256938121631068	Down in EPR_Meteorin
Pgk1	 24644 	phosphoglycerate kinase 1	protein_coding	protein_coding	-0.823185023130591	-1.76930776675806	0.000142202891988303	0.0271553522599435	Down in EPR_Meteorin
Pkm	 25630 	pyruvate kinase M1/2	protein_coding	protein_coding	-1.10981298410472	-2.15817669142237	3.08957850767556e-10	6.65848305239906e-07	Down in EPR_Meteorin
Plat	 25692 	plasminogen activator, tissue type	protein_coding	protein_coding	0.899294058674507	1.86515310071854	3.48768647751074e-07	0.000350768254664847	Up in EPR_Meteorin
Plcd4	 140693 	phospholipase C, delta 4	protein_coding	protein_coding	-1.91975499682399	-3.78358799109036	1.52825110803982e-09	2.88189952698609e-06	Down in EPR_Meteorin
Prdm8	 305198 	PR/SET domain 8	protein_coding	protein_coding	-1.39717289228153	-2.63384946519263	0.000361450615849736	0.0495713090064465	Down in EPR_Meteorin
Psat1	 293820 	phosphoserine aminotransferase 1	protein_coding	protein_coding	-0.933080952036236	-1.90934916205461	0.000171915019492393	0.0298104595869223	Down in EPR_Meteorin
Ptprn	 116660 	protein tyrosine phosphatase, receptor type, N	protein_coding	protein_coding	-3.67430670973706	-12.7666376716621	2.52713940559357e-50	1.90622125363923e-46	Down in EPR_Meteorin
Pygm	 24701 	glycogen phosphorylase, muscle associated	protein_coding	protein_coding	-0.947518538033344	-1.92855265744704	0.000366024980190548	0.0497464220824739	Down in EPR_Meteorin
Rbp3	 24711 	retinol binding protein 3	protein_coding	protein_coding	-2.10913826218523	-4.31433517578101	0.0002346090526598	0.036748782172836	Down in EPR_Meteorin
Rbp4	 25703 	retinol binding protein 4	protein_coding	protein_coding	-2.0950676151811	-4.27246186613215	1.57203322705356e-06	0.00124819438228052	Down in EPR_Meteorin
Resf1	 316982 	retroelement silencing factor 1	NA	NA	1.38926091002829	2.61944452738902	1.73054374813726e-07	0.000186478449888562	Up in EPR_Meteorin
Rgcc	 117183 	regulator of cell cycle	protein_coding	protein_coding	1.37633220602548	2.59607525725798	0.00031288298931056	0.0449538359689439	Up in EPR_Meteorin
RGD1562690	 500965 	similar to L-lactate dehydrogenase A chain (LDH-A	NA	NA	-1.46826310361156	-2.76688580766805	1.99675529776101e-06	0.00143443097247727	Down in EPR_Meteorin
Rgr	 306307 	retinal G protein coupled receptor	protein_coding	protein_coding	0.906806140832135	1.87489024148114	8.13795795284622e-05	0.0191826927619747	Up in EPR_Meteorin
Rho	 24717 	rhodopsin	protein_coding	protein_coding	-2.10752976561398	-4.30952769671187	8.49887491315091e-05	0.0194263677181507	Down in EPR_Meteorin
Rundc3a	 303569 	RUN domain containing 3A	protein_coding	protein_coding	1.49232219741349	2.81341465020736	0.000123607236728835	0.0245360364906738	Up in EPR_Meteorin
Scd	 246074 	stearoyl-CoA desaturase	NA	NA	2.26212197354447	4.79696520041022	6.42863453762609e-15	1.93964761269254e-11	Up in EPR_Meteorin
Sh2d5	 366489 	SH2 domain containing 5	protein_coding	protein_coding	-2.80770687056976	-7.00170787314347	9.98984977020734e-07	0.000941917960208425	Down in EPR_Meteorin
Slc22a8	 83500 	solute carrier family 22 member 8	protein_coding	protein_coding	0.862065970224549	1.81763934632774	0.000217055633387358	0.0358784801093923	Up in EPR_Meteorin
Slc35c2	 311637 	solute carrier family 35 member C2	protein_coding	protein_coding	-0.772091056683933	-1.70774321076149	0.00014695386919349	0.0277118258831623	Down in EPR_Meteorin
Slco1c1	 84511 	solute carrier organic anion transporter family, men	protein_coding	protein_coding	1.12646207206015	2.18322689466634	1.36145308909223e-06	0.00114104896122474	Up in EPR_Meteorin
Sprr1a	 499660 	small proline-rich protein 1A	protein_coding	protein_coding	-7.21522795787629	-148.593578213759	8.39016116761716e-05	0.0194263677181507	Down in EPR_Meteorin
Tdrd7	 85425 	tudor domain containing 7	protein_coding	protein_coding	-1.03246958941396	-2.04552275944139	2.94949011668425e-05	0.00946723572346779	Down in EPR_Meteorin
Tfrc	 64678 	transferrin receptor	protein_coding	protein_coding	-1.24858554115358	-2.37608350845406	0.000327269519422224	0.0465772450000346	Down in EPR_Meteorin
Timp1	 116510 	TIMP metallopeptidase inhibitor 1	protein_coding	protein_coding	-1.35504983297505	-2.55805952046215	0.000137944598011843	0.0266799000718804	Down in EPR_Meteorin
Tk1	 24834 	thymidine kinase 1	protein_coding	protein_coding	-1.50211610283921	-2.83257882287758	3.85082730338574e-05	0.0113693072247535	Down in EPR_Meteorin
Tmem140	 362334 	transmembrane protein 140	protein_coding	protein_coding	0.773138848778637	1.70898395099106	0.000235251342268344	0.036748782172836	Up in EPR_Meteorin
Tmem40	 680858 	transmembrane protein 40	protein_coding	protein_coding	-7.2978767358112	-157.354730010697	5.07585961436851e-05	0.0139226214804297	Down in EPR_Meteorin
Tmem9	 289046 	transmembrane protein 9	protein_coding	protein_coding	-0.821362709267411	-1.76707430887457	4.58300050959639e-05	0.0128035454977354	Down in EPR_Meteorin
Tpm4	 24852 	tropomyosin 4	protein_coding	protein_coding	0.736848414191122	1.66653130054858	9.70169706342263e-05	0.0211082284822366	Up in EPR_Meteorin
Uchl1	 29545 	ubiquitin C-terminal hydrolase L1	protein_coding	protein_coding	-1.54441369233867	-2.91685504497028	8.85906415076014e-05	0.0199474390713981	Down in EPR_Meteorin
Ulbp1	 102547056 	UL16 binding protein 1	NA	NA	1.71351029944403	3.27957825363538	0.000347041796337238	0.0484765975883665	Up in EPR_Meteorin
Vwa1	 298683 	von Willebrand factor A domain containing 1	protein_coding	protein_coding	1.03982648786482	2.05598036681956	2.86614084131257e-06	0.00169674563960406	Up in EPR_Meteorin
Zbtb16	 353227 	zinc finger and BTB domain containing 16	protein_coding	protein_coding	2.21339423669679	4.63765093777963	0.000121026128784055	0.0243440023844834	Up in EPR_Meteorin

Supplementary Table 2 DE genes Retina

FeatureID	EntrezID	Gene Name	TranscriptType	GeneBiotype	logFC	FC	PValue	FDR	Status
AABR07014239.1	 NA 	NA	NA	NA	-1.07465455840786	-2.10621769483329	7.31024163971974e-15	2.55036055205723e-11	Down in NR_Meteorin
AABR07014242.1	 NA 	NA	NA	NA	0.768792833026938	1.70384350541992	2.7698036646382e-08	3.51387364909328e-05	Up in NR_Meteorin
AABR07024972.1	 NA 	NA	NA	NA	1.67933893520076	3.20281159711191	0.000567776569275039	0.0420219003848482	Up in NR_Meteorin
AABR07035375.1	 NA 	NA	NA	NA	1.60588973722203	3.04383411761896	1.78214815872214e-08	2.76331972832972e-05	Up in NR_Meteorin
AABR07039446.1	 NA 	NA	NA	NA	-8.08227145627535	-271.022995747731	0.000144208645287649	0.0218742570107515	Down in NR_Meteorin
AABR07044460.2	 NA 	NA	NA	NA	-0.991484229785725	-1.98822940897547	0.00067526805446969	0.045523505797703	Down in NR_Meteorin
AABR07045632.1	 NA 	NA	NA	NA	-9.31186184662242	-635.550012081412	2.042710466315e-22	1.42530122787129e-18	Down in NR_Meteorin
AABR07059380.1	 NA 	NA	NA	NA	0.372298065538701	1.29441305685208	8.66952937513557e-05	0.0151229103037521	Up in NR_Meteorin
AABR07073045.1	 NA 	NA	NA	NA	-7.96466103798004	-249.805431935087	7.22557257777738e-08	7.75637425560641e-05	Down in NR_Meteorin
AC117058.1	 NA 	NA	NA	NA	-8.60207039186429	-388.580687897545	4.48878884419861e-05	0.00921191887070465	Down in NR_Meteorin
Acaca	 60581 	acetyl-CoA carboxylase alpha	protein_coding	protein_coding	0.418315894071401	1.33636665865055	1.11569102546635e-05	0.00389236706509575	Up in NR_Meteorin
Adgrl2	 171447 	adhesion G protein-coupled receptor L2	NA	NA	0.311895013233584	1.24133715454412	0.000434447834287602	0.0365224067920692	Up in NR_Meteorin
Ank3	 361833 	ankyrin 3	protein_coding	protein_coding	0.345656035028389	1.2707286881491	0.000684854711190992	0.0459478244936072	Up in NR_Meteorin
Ankib1	 368062 	ankyrin repeat and IBR domain containing 1	protein_coding	protein_coding	0.318624236687007	1.24714070023792	0.000345722048588974	0.032408929264027	Up in NR_Meteorin
Ankrd44	 301415 	ankyrin repeat domain 44	protein_coding	protein_coding	0.468555366598713	1.38372319183786	3.00810170311046e-05	0.00711492529947567	Up in NR_Meteorin
Arhgef12	 367072 	Rho guanine nucleotide exchange factor 12	protein_coding	protein_coding	0.320071170457377	1.24839213256625	0.000510481575823148	0.0401345106702439	Up in NR_Meteorin
Atp5f1e	 245958 	ATP synthase F1 subunit epsilon	NA	NA	-0.382176316481174	-1.30330642097242	2.65454985994789e-05	0.00686004505473569	Down in NR_Meteorin
Atxn1	 25049 	ataxin 1	protein_coding	protein_coding	0.369635377728673	1.29202624666187	2.88420424451691e-05	0.00711492529947567	Up in NR_Meteorin
Bace2	 288227 	beta-secretase 2	protein_coding	protein_coding	-1.09144042490347	-2.13086681699125	0.000222855181261897	0.0274420559576967	Down in NR_Meteorin
Baz2b	 317627 	bromodomain adjacent to zinc finger domain, 2B	protein_coding	protein_coding	0.337184431795428	1.2632887483093	0.000363431447510383	0.0327718983076797	Up in NR_Meteorin
Bdp1	 294687 	B double prime 1, subunit of RNA polymerase III transcription initiation factor IIIB	protein_coding	protein_coding	0.330344963922424	1.25731397589507	5.32038473041473e-05	0.010457178720132	Up in NR_Meteorin
Bod1l1	 207118 	biorientation of chromosomes in cell division 1-like 1	protein_coding	protein_coding	0.320983782745182	1.24918208358804	0.000569124985506005	0.0420219003848482	Up in NR_Meteorin
Brwd1	 304061 	bromodomain and WD repeat domain containing 1	protein_coding	protein_coding	0.405427036003877	1.32448089713767	4.35032823842329e-05	0.00919830766169652	Up in NR_Meteorin
Bsn	 29138 	bassoon (presynaptic cytomatrix protein)	protein_coding	protein_coding	0.364404411085882	1.2873500620678	0.000113258098777463	0.018815675814756	Up in NR_Meteorin
Cacna1d	 29716 	calcium voltage-gated channel subunit alpha1 D	protein_coding	protein_coding	0.42567959637143	1.34320508437832	2.96672209286873e-06	0.00159233103099935	Up in NR_Meteorin
Camk1d	 307124 	calcium/calmodulin-dependent protein kinase ID	protein_coding	protein_coding	0.308920476466337	1.23878041245114	0.000774142930944992	0.0496757030629073	Up in NR_Meteorin
Carmil1	 306941 	capping protein regulator and myosin 1 linker 1	NA	NA	0.381487383878426	1.30268419943015	0.000116644433025749	0.0189444817409312	Up in NR_Meteorin
Cc2d2a	 498386 	coiled-coil and C2 domain containing 2A	protein_coding	protein_coding	0.563903317989265	1.4782633623374	2.91568125573528e-05	0.00711492529947567	Up in NR_Meteorin
Ccdc141	 311134 	coiled-coil domain containing 141	protein_coding	protein_coding	0.373667992895205	1.2956427651266	0.00034186237297224	0.032408929264027	Up in NR_Meteorin
Ccdc88a	 305605 	coiled coil domain containing 88A	protein_coding	protein_coding	0.337387695978718	1.26346674812109	0.000192826650308106	0.0258739990870156	Up in NR_Meteorin
Ccnj	 294053 	cyclin J	protein_coding	protein_coding	1.39301728408179	2.62627371450243	0.000506400210587736	0.0401345106702439	Up in NR_Meteorin
Cd63	 29186 	Cd63 molecule	protein_coding	protein_coding	-0.303893033923883	-1.23447107582244	0.000625040073078161	0.0438313277377172	Down in NR_Meteorin
Celf2	 29428 	CUGBP, Elav-like family member 2	protein_coding	protein_coding	0.300276769714996	1.2313806213898	0.00034538703565667	0.032408929264027	Up in NR_Meteorin
Cep350	 246304 	centrosomal protein 350	protein_coding	protein_coding	0.366196174889846	1.28894988748953	0.000222154846674757	0.0274420559576967	Up in NR_Meteorin
Ciart	 365871 	circadian associated repressor of transcription	protein_coding	protein_coding	-0.523029144703751	-1.43696920528374	0.0004817756207635	0.0394105798778309	Down in NR_Meteorin
Clic6	 304081 	chloride intracellular channel 6	protein_coding	protein_coding	-0.652677495342441	-1.57208311651675	2.80925489079366e-05	0.00711492529947567	Down in NR_Meteorin
Clu	 24854 	clusterin	protein_coding	protein_coding	-0.378861303051147	-1.30031513162457	6.00007960099178e-05	0.0114700151824439	Down in NR_Meteorin
Cmpk2	 314004 	cytidine/uridine monophosphate kinase 2	protein_coding	protein_coding	-0.444969317359508	-1.36128516494263	1.86287498235655e-06	0.00113027914690373	Down in NR_Meteorin
Col11a2	 294279 	collagen type XI alpha 2 chain	protein_coding	protein_coding	0.567678336394552	1.48213651547531	0.000205735444729214	0.0267568960811513	Up in NR_Meteorin
Col1a1	 29393 	collagen type I alpha 1 chain	protein_coding	protein_coding	2.20013736493703	4.59523093002297	0.000236789249716775	0.028003338811844	Up in NR_Meteorin
Col8a2	 313592 	collagen type VIII alpha 2 chain	protein_coding	protein_coding	-0.875246142319176	-1.83432101845859	0.000147071632884167	0.0220686520096619	Down in NR_Meteorin
Cope	 290659 	COPI coat complex subunit epsilon	protein_coding	protein_coding	-0.330837725699691	-1.25774349292434	0.000293930499495287	0.0303106632880206	Down in NR_Meteorin
Crip2	 338401 	cysteine-rich protein 2	protein_coding	protein_coding	-0.316351074338268	-1.24517720764364	0.00038650449900158	0.0337104392722941	Down in NR_Meteorin
Dbp	 24309 	D-box binding PAR bZIP transcription factor	protein_coding	protein_coding	-0.460895684984985	-1.37639607676788	2.86380407362624e-06	0.00159233103099935	Down in NR_Meteorin
Dhps	 288923 	deoxyhypusine synthase	protein_coding	protein_coding	-0.303411001000733	-1.2340586835389	0.000383340334815519	0.0337104392722941	Down in NR_Meteorin
Dio1	 25430 	iodothyronine deiodinase 1	NA	NA	-0.547143293643342	-1.46118950191798	1.99325791472629e-07	0.000154532856666696	Down in NR_Meteorin
Dst	 316313 	dystonin	protein_coding	protein_coding	0.339343446213991	1.26518069387265	0.000661149836964338	0.044788087256492	Up in NR_Meteorin
Egr1	 24330 	early growth response 1	protein_coding	protein_coding	-1.44858713418835	-2.72940623132608	3.9006584212214e-23	5.44336882681447e-19	Down in NR_Meteorin
Eml5	 444982 	EMAP like 5	protein_coding	protein_coding	0.374495642026041	1.29638626616293	0.000518670434738117	0.0401345106702439	Up in NR_Meteorin
Emsy	 361607 	EMSY transcriptional repressor, BRCA2 interacting	NA	NA	0.30927252592663	1.23908273912315	0.000265530377325001	0.029151829094757	Up in NR_Meteorin
Ep300	 170915 	E1A binding protein p300	protein_coding	protein_coding	0.384040286735813	1.30499138859142	5.05224250884681e-06	0.00243117393830887	Up in NR_Meteorin
Epb41l5	 304733 	erythrocyte membrane protein band 4.1 like 5	NA	NA	0.312783009826053	1.24210144806583	0.000644192786011573	0.04425724491749	Up in NR_Meteorin
Etnppl	 687071 	ethanolamine-phosphate phospho-lyase	protein_coding	protein_coding	0.906966335169153	1.87509843757686	0.000384048054174654	0.0337104392722941	Up in NR_Meteorin
Fkbp2	 293702 	FKBP prolyl isomerase 2	protein_coding	protein_coding	-0.487244354881276	-1.40176485135087	9.02806140979086e-05	0.0152792331682166	Down in NR_Meteorin
Flnb	 306204 	filamin B	protein_coding	protein_coding	0.365018394599285	1.28789805031731	3.41121091616327e-05	0.00767797553791265	Up in NR_Meteorin
Fmn1	 296512 	formin 1	protein_coding	protein_coding	0.458959657598779	1.37455025818106	0.000121741353825729	0.0193056885527051	Up in NR_Meteorin
Fn1	 25661 	fibronectin 1	protein_coding	protein_coding	0.761354038073094	1.6950807925171	0.000755278758567614	0.0490782763261071	Up in NR_Meteorin
Fos	 314322 	Fos proto-oncogene, AP-1 transcription factor subunit	protein_coding	protein_coding	-1.01642170292009	-2.02289537203994	5.73598713025248e-11	1.14351000575248e-07	Down in NR_Meteorin
Frzb	 295691 	frizzled-related protein	protein_coding	protein_coding	-0.33521877170785	-1.26156869985683	0.000167512437353825	0.0237510433592312	Down in NR_Meteorin
Fundc2	 361288 	FUN14 domain containing 2	protein_coding	protein_coding	-0.30519360814553	-1.23558444012379	0.000299151746383066	0.0304719899326692	Down in NR_Meteorin
Gfap	 24387 	glial fibrillary acidic protein	protein_coding	protein_coding	0.472292243987285	1.38731196581317	0.000131568970536852	0.0206297189195705	Up in NR_Meteorin
Gja1	 24392 	gap junction protein, alpha 1	protein_coding	protein_coding	-0.8230666117962	-1.76916255415932	7.06976347132545e-06	0.00298965300734384	Down in NR_Meteorin
Gpx4	 29328 	glutathione peroxidase 4	protein_coding	protein_coding	-0.453712724216658	-1.36956023995183	1.14548229884798e-05	0.00389883060498134	Down in NR_Meteorin
Gramd1b	 300644 	GRAM domain containing 1B	protein_coding	protein_coding	0.32549535248066	1.25309461753132	0.000364001736846317	0.0327718983076797	Up in NR_Meteorin
Grm1	 24414 	glutamate metabotropic receptor 1	protein_coding	protein_coding	0.358328480167285	1.28193976952147	0.00032783811673729	0.0315515925453026	Up in NR_Meteorin
Helz	 287773 	helicase with zinc finger	protein_coding	protein_coding	0.309502710201207	1.2392804525054	0.000305969465761553	0.0305413121790465	Up in NR_Meteorin
Hipk2	 362342 	homeodomain interacting protein kinase 2	protein_coding	protein_coding	0.418288151102122	1.336340960619	0.000172377809686318	0.0240553233417257	Up in NR_Meteorin
Hivep2	 29721 	HIVEP zinc finger 2	protein_coding	protein_coding	0.316965753243309	1.24570784455231	0.000268345079913489	0.029151829094757	Up in NR_Meteorin
Hopx	 171160 	HOP homeobox	protein_coding	protein_coding	-0.375016620807048	-1.29685449519185	9.08761270485113e-05	0.0152792331682166	Down in NR_Meteorin
Hydin	 292017 	Hydin, axonemal central pair apparatus protein	protein_coding	protein_coding	0.572627230616478	1.48722943642391	0.000228084353200025	0.0274420559576967	Up in NR_Meteorin
Igf1r	 25718 	insulin-like growth factor 1 receptor	protein_coding	protein_coding	0.381280313794574	1.30249723852622	8.46631606102002e-06	0.00322108144520942	Up in NR_Meteorin
Ip6k2	 59268 	inositol hexakisphosphate kinase 2	protein_coding	protein_coding	0.428666892233628	1.34598925429039	0.000628942385380386	0.0438844549399164	Up in NR_Meteorin
Iqgap2	<a href='https://www.ncbi.nlm.nih.gov/gene/?term=100360623' target="_blank"> 10036062	IQ motif containing GTPase activating protein 2	protein_coding	protein_coding	-0.778926596888353	-1.71585375743878	1.79274770278143e-05	0.00532293493453509	Down in NR_Meteorin
Jag1	 29146 	jagged canonical Notch ligand 1	protein_coding	protein_coding	0.299704587537325	1.23089234470992	0.000588416960337711	0.0429914067094909	Up in NR_Meteorin
Jak3	 25326 	Janus kinase 3	protein_coding	protein_coding	-0.736226192963998	-1.66581269575697	0.000367742184465532	0.032896424257798	Down in NR_Meteorin
Kat6b	 688634 	lysine acetyltransferase 6B	protein_coding	protein_coding	0.311708111628859	1.24117634933449	0.00034603586243927	0.032408929264027	Up in NR_Meteorin
Kcna2	 25468 	potassium voltage-gated channel subfamily A member 2	protein_coding	protein_coding	0.425327946793918	1.34287772486935	3.17417799558357e-05	0.00726158261120798	Up in NR_Meteorin
Kif21b	 289397 	kinesin family member 21B	protein_coding	protein_coding	0.34383408439531	1.26912492326841	0.000356526178589332	0.0327323869882508	Up in NR_Meteorin
Kitlg	 60427 	KIT ligand	protein_coding	protein_coding	0.327582966134537	1.25490918745958	0.000276674872470925	0.029151829094757	Up in NR_Meteorin
Klf9	 117560 	Kruppel-like factor 9	protein_coding	protein_coding	0.333551084704114	1.26011122885387	0.000493786291215862	0.0397348963879807	Up in NR_Meteorin
Kmt2a	 315606 	lysine methyltransferase 2A	protein_coding	protein_coding	0.427548520049234	1.3449462524138	3.47147489447809e-05	0.00768959240514949	Up in NR_Meteorin
Kmt2c	 502710 	lysine methyltransferase 2C	protein_coding	protein_coding	0.351612802544855	1.27598626751965	0.000168495398965524	0.0237510433592312	Up in NR_Meteorin
Krt18	 294853 	keratin 18	protein_coding	protein_coding	-1.11409922456803	-2.16459815899645	3.75538025929258e-06	0.00194097524142326	Down in NR_Meteorin
Krt19	 360626 	keratin 19	protein_coding	protein_coding	-0.495785407593799	-1.41008820255396	1.97864563707158e-07	0.000154532856666696	Down in NR_Meteorin
Krt8	 25626 	keratin 8	protein_coding	protein_coding	-1.06355216998768	-2.0900713162566	0.00031332545690784	0.0307919489517529	Down in NR_Meteorin
Lamb1	 298941 	laminin subunit beta 1	protein_coding	protein_coding	0.460871713808416	1.37637320737529	0.00052055510077493	0.0401345106702439	Up in NR_Meteorin
Lcn2	 170496 	lipocalin 2	protein_coding	protein_coding	-1.56963370492329	-2.96829340656778	3.36122865102343e-08	3.90882881875266e-05	Down in NR_Meteorin
Lcor	 365462 	ligand dependent nuclear receptor corepressor	protein_coding	protein_coding	0.576510364914803	1.49123783081482	1.42602146341395e-06	0.000947625215330556	Up in NR_Meteorin
LOC100360117	<a href='https://www.ncbi.nlm.nih.gov/gene/?term=100360117' target="_blank"> 10036011	ribosomal protein L8-like	protein_coding	protein_coding	-0.330640661193591	-1.25757170355373	0.000407532670198715	0.0349777560134473	Down in NR_Meteorin
LOC100361025	<a href='https://www.ncbi.nlm.nih.gov/gene/?term=100361025' target="_blank"> 10036102	protein arginine methyltransferase 1-like	NA	NA	-0.35689272670053	-1.28066463245295	1.73532805558648e-05	0.00526445717732813	Down in NR_Meteorin
LOC100362366	<a href='https://www.ncbi.nlm.nih.gov/gene/?term=100362366' target="_blank"> 10036236	40S ribosomal protein S17-like	protein_coding	protein_coding	-0.330834099633417	-1.25774033171882	0.000281614189770909	0.0293278061063659	Down in NR_Meteorin
LOC100911660	<a href='https://www.ncbi.nlm.nih.gov/gene/?term=100911660' target="_blank"> 10091166	lymphocyte-specific helicase-like	protein_coding	protein_coding	0.549246749698726	1.46332147682022	1.72625895192248e-05	0.00526445717732813	Up in NR_Meteorin
LOC100911847	<a href='https://www.ncbi.nlm.nih.gov/gene/?term=100911847' target="_blank"> 10091184	40S ribosomal protein S14-like	protein_coding	protein_coding	-0.391892255514818	-1.31211325865883	1.48902264311646e-05	0.00472257067833869	Down in NR_Meteorin
LOC100911991	<a href='https://www.ncbi.nlm.nih.gov/gene/?term=100911991' target="_blank"> 10091199	elongation factor 1-alpha 1-like	protein_coding	protein_coding	-0.508250164360918	-1.42232402121684	0.000555851773060309	0.0419358895341454	Down in NR_Meteorin
LOC100912282	<a href='https://www.ncbi.nlm.nih.gov/gene/?term=100912282' target="_blank"> 10091228	calcium-binding and coiled-coil domain-containing protein 1-like	protein_coding	protein_coding	-0.849935556800484	-1.80242041178731	0.000243373599603945	0.0285401561552358	Down in NR_Meteorin
LOC102555083	<a href='https://www.ncbi.nlm.nih.gov/gene/?term=102555083' target="_blank"> 10255508	zinc finger protein 250-like	protein_coding	protein_coding	-8.99626778573873	-510.677181204786	8.54030909872794e-06	0.00322108144520942	Down in NR_Meteorin
LOC103689943	<a href='https://www.ncbi.nlm.nih.gov/gene/?term=103689943' target="_blank"> 10368994	meiosis arrest female protein 1-like	NA	NA	0.364646440673225	1.28756604876791	7.71519849084921e-06	0.00316663514528826	Up in NR_Meteorin
LOC103689951	<a href='https://www.ncbi.nlm.nih.gov/gene/?term=103689951' target="_blank"> 10368995	liprin-alpha-1-like	NA	NA	-7.65868873100645	-202.066833316724	2.41255598249691e-08	3.36672187357444e-05	Down in NR_Meteorin
LOC103690160	<a href='https://www.ncbi.nlm.nih.gov/gene/?term=103690160' target="_blank"> 10369016	SH3 and multiple ankyrin repeat domains protein 2-like	NA	NA	1.24294938748581	2.36681900948994	0.000271356925512202	0.029151829094757	Up in NR_Meteorin
LOC103690996	<a href='https://www.ncbi.nlm.nih.gov/gene/?term=103690996' target="_blank"> 10369099	uncharacterized LOC103690996	NA	NA	-0.591395119507516	-1.50670305989752	0.000493839837369784	0.0397348963879807	Down in NR_Meteorin
LOC108348055	<a href='https://www.ncbi.nlm.nih.gov/gene/?term=108348055' target="_blank"> 10834805	B9 domain-containing protein 1	NA	NA	-2.50599602172236	-5.68041377022976	0.000756132526701041	0.0490782763261071	Down in NR_Meteorin
LOC498555	 498555 	similar to 60S acidic ribosomal protein P2	protein_coding	protein_coding	-0.432296263002406	-1.34937960620708	6.21343875664888e-06	0.00279704960803339	Down in NR_Meteorin
LOC679894	 679894 	similar to THO complex subunit 2 (Tho2)	protein_coding	protein_coding	0.425894678150233	1.34340534879092	0.000607752987574136	0.0438313277377172	Up in NR_Meteorin
Lrat	 64047 	lecithin retinol acyltransferase	protein_coding	protein_coding	-1.2102009211006	-2.3136985693019	1.49687339615977e-07	0.00013055542652131	Down in NR_Meteorin
Macf1	 362587 	microtubule-actin crosslinking factor 1	protein_coding	protein_coding	0.502370265068067	1.41653894371916	1.31625140211917e-07	0.00013055542652131	Up in NR_Meteorin
Map1b	 29456 	microtubule-associated protein 1B	protein_coding	protein_coding	0.277621389840857	1.21219465796308	0.000325641849038445	0.0315515925453026	Up in NR_Meteorin
Mast4	<a href='https://www.ncbi.nlm.nih.gov/gene/?term=100912235' target="_blank"> 10091223	microtubule associated serine/threonine kinase family member 4	protein_coding	protein_coding	1.4114502160453	2.66004419615788	2.4662615414924e-05	0.00661859227144739	Up in NR_Meteorin
Mbd3	 362834 	methyl-CpG binding domain protein 3	protein_coding	protein_coding	-0.315839523255068	-1.2447357707385	0.000260230000757483	0.029151829094757	Down in NR_Meteorin
Mbd6	 362892 	methyl-CpG binding domain protein 6	protein_coding	protein_coding	0.369867229164274	1.29223390122745	0.000139399725864423	0.0213771777410771	Up in NR_Meteorin
Mbnl1	 282635 	muscleblind-like splicing regulator 1	protein_coding	protein_coding	0.311713052449749	1.24118060001836	0.000606119035216262	0.0438313277377172	Up in NR_Meteorin
Mea1	 685131 	male-enhanced antigen 1	protein_coding	protein_coding	-0.311099337329293	-1.24065272040102	0.000120578571973782	0.0193056885527051	Down in NR_Meteorin
Med13	 303403 	mediator complex subunit 13	protein_coding	protein_coding	0.322667997666048	1.25064124132611	0.000515372828254053	0.0401345106702439	Up in NR_Meteorin
Mest	 58827 	mesoderm specific transcript	protein_coding	protein_coding	-0.456993494696367	-1.37267824165237	8.64526589470885e-05	0.0151229103037521	Down in NR_Meteorin
Mfap4	 287382 	microfibril associated protein 4	NA	NA	-8.19310428405455	-292.664568125129	0.000116748508041568	0.0189444817409312	Down in NR_Meteorin
Mfrp	 315597 	membrane frizzled-related protein	protein_coding	protein_coding	-0.917457565961503	-1.88878379103097	1.5088194037027e-06	0.00095707158084869	Down in NR_Meteorin
Mga	 499874 	MAX dimerization protein MGA	protein_coding	protein_coding	0.358659176445309	1.28223365096509	0.000133965107497176	0.0207720341680343	Up in NR_Meteorin
Mib1	 307594 	mindbomb E3 ubiquitin protein ligase 1	protein_coding	protein_coding	0.349351445194726	1.27398778508835	0.000748738485867494	0.0490546740388774	Up in NR_Meteorin
Mmp3	 171045 	matrix metallopeptidase 3	protein_coding	protein_coding	-3.85952694400226	-14.5155460943822	6.5252144531836e-06	0.00284560524044304	Down in NR_Meteorin
Myo9a	 171296 	myosin IXA	protein_coding	protein_coding	0.301471734947239	1.23240098019813	0.000775002442257546	0.0496757030629073	Up in NR_Meteorin
Myoc	 81523 	myocilin	protein_coding	protein_coding	-0.841180720454118	-1.7915157419103	0.000776015999119583	0.0496757030629073	Down in NR_Meteorin
Ndufb2	 362344 	NADH:ubiquinone oxidoreductase subunit B2	protein_coding	protein_coding	-0.340206031463976	-1.26593736973994	0.000559474790359256	0.0419358895341454	Down in NR_Meteorin
NEWGENE_62135	 NA 	NA	NA	NA	8.36578171255772	329.876389573604	5.22886006450769e-05	0.0104241060286007	Up in NR_Meteorin
Nktr	<a href='https://www.ncbi.nlm.nih.gov/gene/?term=100364165' target="_blank"> 10036416	natural killer cell triggering receptor	protein_coding	protein_coding	0.453635711766587	1.3694871334591	7.97902584865769e-05	0.0144606890542881	Up in NR_Meteorin
Nr4a1	 79240 	nuclear receptor subfamily 4, group A, member 1	protein_coding	protein_coding	-0.683236209722364	-1.60573766029182	2.7300070564644e-07	0.000200511834068214	Down in NR_Meteorin
Nsd3	 290831 	nuclear receptor binding SET domain protein 3	NA	NA	0.287412044932323	1.22044903024176	0.000413750079941355	0.0352065997901318	Up in NR_Meteorin
Nudt14	 299346 	nudix hydrolase 14	protein_coding	protein_coding	-0.562177514185391	-1.47649606768703	0.000355609826613785	0.0327323869882508	Down in NR_Meteorin
Nxnl1	 306342 	nucleoredoxin-like 1	protein_coding	protein_coding	-0.343960244759614	-1.2692359101777	0.000201467387417201	0.0265233716170476	Down in NR_Meteorin
Pard3b	 301455 	par-3 family cell polarity regulator beta	protein_coding	protein_coding	0.943456173457822	1.92312984535799	0.000655904093028688	0.0446494713083676	Up in NR_Meteorin
Pask	 301617 	PAS domain containing serine/threonine kinase	protein_coding	protein_coding	0.653179271248103	1.57262998928129	0.00027783541881782	0.029151829094757	Up in NR_Meteorin
Pclo	 56768 	piccolo (presynaptic cytomatrix protein)	protein_coding	protein_coding	0.452310457957682	1.36822970575923	0.000646970832186884	0.04425724491749	Up in NR_Meteorin
Pcnx2	 307949 	pecanex 2	NA	NA	0.372121420745856	1.2942545775323	0.000534997091969992	0.040797182614433	Up in NR_Meteorin
Pde8a	 308776 	phosphodiesterase 8A	protein_coding	protein_coding	0.432588989706399	1.34965342673692	0.000214261238670366	0.0271819598694996	Up in NR_Meteorin
Phb	 25344 	prohibitin	protein_coding	protein_coding	-0.324046948996012	-1.25183719598348	0.00031604128956791	0.0308416517197216	Down in NR_Meteorin
Pla2r1	 295631 	phospholipase A2 receptor 1	protein_coding	protein_coding	0.467737656704189	1.3829391290145	2.22065845806472e-05	0.00617193369491891	Up in NR_Meteorin
Plekha4	 308584 	pleckstrin homology domain containing A4	protein_coding	protein_coding	-0.980665783930391	-1.97337588472974	4.720590918612e-05	0.00954722409698993	Down in NR_Meteorin
Plxna2	 289392 	plexin A2	protein_coding	protein_coding	0.359984550134038	1.28341215338873	2.19264611738344e-05	0.00617193369491891	Up in NR_Meteorin

Pmel	 362818 	premelanosome protein	protein_coding	protein_coding	-0.544111174398943	-1.45812173767093	0.000420520078230427	0.0355658041921552	Down in NR_Meteorin
Polr2e	 690966 	RNA polymerase II, I and III subunit E	protein_coding	protein_coding	-0.31222924386716	-1.24162476970528	0.000228015722687068	0.0274420559576967	Down in NR_Meteorin
Ppp1r12b	 304813 	protein phosphatase 1, regulatory subunit 12B	protein_coding	protein_coding	0.304806192858674	1.23525268599658	0.000482924339599361	0.0394105798778309	Up in NR_Meteorin
Ppp1r1b	 360616 	protein phosphatase 1, regulatory (inhibitor) subunit 1B	protein_coding	protein_coding	-0.553747297050849	-1.46789349679529	5.22027924145831e-07	0.000364244984072754	Down in NR_Meteorin
Prdm1	 309871 	PR/SET domain 1	protein_coding	protein_coding	0.479099835912828	1.39387369277769	4.42892679514258e-05	0.00921191887070465	Up in NR_Meteorin
Prex2	 312912 	phosphatidylinositol-3,4,5-trisphosphate-dependent Rac exchange factor 2	protein_coding	protein_coding	0.383730129636733	1.30471086580087	0.000153136952481624	0.0222606892904277	Up in NR_Meteorin
Prkcq	 85420 	protein kinase C, theta	protein_coding	protein_coding	-0.766954046847795	-1.70167325594638	7.26354014809729e-05	0.0135150270355597	Down in NR_Meteorin
Psd3	 306380 	pleckstrin and Sec7 domain containing 3	protein_coding	protein_coding	1.11718030315869	2.16922590328071	1.59172812322892e-16	7.40418865321986e-13	Up in NR_Meteorin
Psmb1	 94198 	proteasome 20S subunit beta 1	protein_coding	protein_coding	-0.36127834223301	-1.28456361868369	0.000482336368291065	0.0394105798778309	Down in NR_Meteorin
Psmb4	 58854 	proteasome 20S subunit beta 4	protein_coding	protein_coding	-0.380978267163425	-1.30222457263947	0.000504890028853792	0.0401345106702439	Down in NR_Meteorin
Psmb5	 29425 	proteasome 20S subunit beta 5	protein_coding	protein_coding	-0.331745960787711	-1.25853554279325	0.000221559306840538	0.0274420559576967	Down in NR_Meteorin
Psme4	 498433 	proteasome activator subunit 4	protein_coding	protein_coding	0.329493163378226	1.25657184772879	0.000408554226455887	0.0349777560134473	Up in NR_Meteorin
Ptgds	 25526 	prostaglandin D2 synthase	protein_coding	protein_coding	-0.320857102469633	-1.24907240012854	0.000148947800631688	0.0220996754798312	Down in NR_Meteorin
Rabac1	 83583 	Rab acceptor 1	protein_coding	protein_coding	-0.370395855771721	-1.29270748321827	1.37598117100368e-05	0.00446553889333887	Down in NR_Meteorin
Rbm41	 680581 	RNA binding motif protein 41	protein_coding	protein_coding	0.550939574227868	1.46503951167938	6.15914669090822e-05	0.0116149854150844	Up in NR_Meteorin
Rbp1	 25056 	retinol binding protein 1	protein_coding	protein_coding	-0.403174836613875	-1.3224148555904	3.76338176602518e-05	0.00808968364931059	Down in NR_Meteorin
Rc3h1	 680586 	ring finger and CCCH-type domains 1	processed_trans	processed_trans	0.294393145579427	1.2263690095813	0.000495440485238886	0.0397348963879807	Up in NR_Meteorin
Rev3l	 309812 	REV3 like, DNA directed polymerase zeta catalytic subunit	protein_coding	protein_coding	0.337946871457846	1.26395655125919	0.000301813539554629	0.0305203474238032	Up in NR_Meteorin
Rfx7	 315804 	regulatory factor X, 7	protein_coding	protein_coding	0.314801913804592	1.24384085863151	0.000247607827460307	0.0285509478511214	Up in NR_Meteorin
RGD1565616	 499891 	RGD1565616	protein_coding	protein_coding	0.450410112606859	1.36642863402771	0.000311970622698936	0.0307919489517529	Up in NR_Meteorin
Rictor	 310131 	RPTOR independent companion of MTOR, complex 2	protein_coding	protein_coding	0.33774570190944	1.26378031731192	0.000249603413675157	0.0285509478511214	Up in NR_Meteorin
Rlim	 317241 	ring finger protein, LIM domain interacting	protein_coding	protein_coding	0.543823444536702	1.45783096011869	3.76803609606011e-05	0.00808968364931059	Up in NR_Meteorin
Rnh1	<a href='https://www.ncbi.nlm.nih.gov/gene/?term=100360501' target="_blank"> 10036050	ribonuclease/angiogenin inhibitor 1	protein_coding	protein_coding	-0.375944878562598	-1.29768918483233	0.00019630097570309	0.0260893344374917	Down in NR_Meteorin
Rom1	 309201 	retinal outer segment membrane protein 1	protein_coding	protein_coding	-0.259580561768303	-1.19713060933174	0.000613993838859062	0.0438313277377172	Down in NR_Meteorin
Rpe65	 89826 	retinoid isomerohydrolase RPE65	protein_coding	protein_coding	-1.03189360580065	-2.04470626503518	2.19986578793573e-11	6.13982541412863e-08	Down in NR_Meteorin
Rpl22l1	 361923 	ribosomal protein L22 like 1	protein_coding	protein_coding	-0.447230232801089	-1.3634201716259	2.97016478484131e-05	0.00711492529947567	Down in NR_Meteorin
Rpl39l	 497860 	ribosomal protein L39-like	protein_coding	protein_coding	-2.17931427101172	-4.52938216377576	0.000191967492624062	0.0258739990870156	Down in NR_Meteorin
Rplp1	 140661 	ribosomal protein lateral stalk subunit P1	protein_coding	protein_coding	-0.366628914085167	-1.28933656851577	0.000186975045971908	0.0255807526131174	Down in NR_Meteorin
Rps10	 81773 	ribosomal protein S10	protein_coding	protein_coding	-0.364444928679995	-1.28738621735931	0.000272371676632148	0.029151829094757	Down in NR_Meteorin
Rps15	 29285 	ribosomal protein S15	protein_coding	protein_coding	-0.301787254108504	-1.23267053726754	0.00062272509763584	0.0438313277377172	Down in NR_Meteorin
Rps17	 29286 	ribosomal protein S17	protein_coding	protein_coding	-0.313463689515737	-1.24268762372146	0.000716143886912575	0.0475894663898333	Down in NR_Meteorin
Rps18l1	<a href='https://www.ncbi.nlm.nih.gov/gene/?term=100360679' target="_blank"> 10036067	ribosomal protein S18-like 1	NA	NA	-0.430072301100714	-1.34730109557795	5.8407648277848e-06	0.00271692910572456	Down in NR_Meteorin
Rps19l2	 29287 	ribosomal protein S19-like2	NA	NA	-0.318689475053828	-1.24719709695363	0.000462474062111357	0.0386456618967904	Down in NR_Meteorin
Rxfp1	 295144 	relaxin family peptide receptor 1	protein_coding	protein_coding	1.07295223308413	2.10373390367052	0.000384097976869759	0.0337104392722941	Up in NR_Meteorin
Ryr2	 689560 	ryanodine receptor 2	protein_coding	protein_coding	0.463864051377495	1.37923094745487	0.000208638582956403	0.0267568960811513	Up in NR_Meteorin
Sacs	 305940 	sacsin molecular chaperone	protein_coding	protein_coding	0.443925065144497	1.36030019537657	2.25559740910687e-05	0.00617193369491891	Up in NR_Meteorin
Sag	 25539 	S-antigen visual arrestin	protein_coding	protein_coding	-0.393324653315731	-1.31341665364429	9.57473896331196e-06	0.00351619690086891	Down in NR_Meteorin
Samd11	<a href='https://www.ncbi.nlm.nih.gov/gene/?term=102549710' target="_blank"> 10254971	sterile alpha motif domain containing 11	protein_coding	protein_coding	0.385740768205356	1.30653046784385	8.2785561866157e-05	0.0148111861005413	Up in NR_Meteorin
Sbk1	 113907 	SH3 domain binding kinase 1	protein_coding	protein_coding	0.401229811102028	1.32063319177484	0.000263642426640085	0.029151829094757	Up in NR_Meteorin
Scn2a	 24766 	sodium voltage-gated channel alpha subunit 2	NA	NA	0.319849865973943	1.24820064817109	0.000267429928455698	0.029151829094757	Up in NR_Meteorin
Scn8a	 29710 	sodium voltage-gated channel alpha subunit 8	protein_coding	protein_coding	0.40681657816822	1.32575719503347	8.47387157041246e-06	0.00322108144520942	Up in NR_Meteorin
Sema3c	 296787 	semaphorin 3C	protein_coding	protein_coding	-0.579362541592251	-1.49418889166115	0.000709090483425594	0.0473462090727472	Down in NR_Meteorin
Setx	 362096 	senataxin	protein_coding	protein_coding	0.328551298292934	1.25575176110652	0.000154889466612179	0.0222833248100304	Up in NR_Meteorin
Sf3b5	 680891 	splicing factor 3b, subunit 5	protein_coding	protein_coding	-0.414352351556425	-1.33270027271866	2.58040653427248e-05	0.00679425909165519	Down in NR_Meteorin
Sik1	 59329 	salt-inducible kinase 1	protein_coding	protein_coding	-0.698279591644624	-1.62256873152099	0.000475996600625339	0.0394105798778309	Down in NR_Meteorin
Slc16a8	 65200 	solute carrier family 16 member 8	protein_coding	protein_coding	-0.936220635689893	-1.91350893248161	0.000622766658503228	0.0438313277377172	Down in NR_Meteorin
Slc4a7	 117955 	solute carrier family 4 member 7	protein_coding	protein_coding	0.407145906536683	1.32605986418981	0.000561949934997147	0.0419358895341454	Up in NR_Meteorin
Slc6a13	 171163 	solute carrier family 6 member 13	protein_coding	protein_coding	-1.35655811360958	-2.56073526901992	2.04305701365774e-05	0.00593976263033203	Down in NR_Meteorin
Slc7a8	 84551 	solute carrier family 7 member 8	protein_coding	protein_coding	0.324275657991378	1.25203566420752	0.000228110246584938	0.0274420559576967	Up in NR_Meteorin
Slco1a4	 170698 	solute carrier organic anion transporter family, member 1a4	protein_coding	protein_coding	-0.413462919721852	-1.3318789066622	9.01473298710651e-05	0.0152792331682166	Down in NR_Meteorin
Snip1	 313588 	Smad nuclear interacting protein 1	protein_coding	protein_coding	0.909737304925804	1.87870338104008	0.000150445658945465	0.0220996754798312	Up in NR_Meteorin
Srcap	 361652 	Snf2-related CREBBP activator protein	protein_coding	protein_coding	0.353888264413288	1.2780003792542	0.000398515768693754	0.0345421587088281	Up in NR_Meteorin
Srrm2	 302969 	serine/arginine repetitive matrix 2	protein_coding	protein_coding	0.317080099566899	1.24580658181369	0.000350430349129601	0.0326017034806905	Up in NR_Meteorin
Stard9	 691920 	StAR-related lipid transfer domain containing 9	NA	NA	0.716988166937513	1.6437468924338	4.15790396171627e-06	0.00207226963520538	Up in NR_Meteorin
Stra6	 363071 	signaling receptor and transporter of retinol STRA6	protein_coding	protein_coding	-0.669121226537488	-1.59010410928652	0.000514019003568482	0.0401345106702439	Down in NR_Meteorin
Taok1	 286993 	TAO kinase 1	protein_coding	protein_coding	0.30965342238563	1.23940992159965	0.000531231060016077	0.0407325793545294	Up in NR_Meteorin
Tbca	 366995 	tubulin folding cofactor A	protein_coding	protein_coding	-0.323690994636241	-1.251528369843	0.000574168051748366	0.0421711324323602	Down in NR_Meteorin
Tet1	 309902 	tet methylcytosine dioxygenase 1	protein_coding	protein_coding	0.560141993703993	1.47441432574645	0.000738281477166227	0.048828047459027	Up in NR_Meteorin
Tet2	 310859 	tet methylcytosine dioxygenase 2	protein_coding	protein_coding	0.306079693242876	1.23634355359754	0.000306397972416088	0.0305413121790465	Up in NR_Meteorin
Tet3	 680576 	tet methylcytosine dioxygenase 3	protein_coding	protein_coding	0.371780919592041	1.29394914695778	0.000362387448153103	0.0327718983076797	Up in NR_Meteorin
Tex10	 298065 	testis expressed 10	protein_coding	protein_coding	0.413551761756058	1.33196092709717	3.15340784052571e-05	0.00726158261120798	Up in NR_Meteorin
Tf	 24825 	transferrin	protein_coding	protein_coding	-0.722115974039452	-1.64959969839077	3.326609219689e-11	7.73713861012666e-08	Down in NR_Meteorin
Tnrc6a	 308971 	trinucleotide repeat containing adaptor 6A	protein_coding	protein_coding	0.319076519538749	1.24753173837262	0.000618617507762675	0.0438313277377172	Up in NR_Meteorin
Trip11	 314393 	thyroid hormone receptor interactor 11	protein_coding	protein_coding	0.315885479050702	1.24477542134529	0.000248211044276867	0.0285509478511214	Up in NR_Meteorin
Ttbk2	 311349 	tau tubulin kinase 2	protein_coding	protein_coding	0.485508946609614	1.40007969146905	1.45564028775535e-07	0.00013055542652131	Up in NR_Meteorin
Ttr	 24856 	transthyretin	protein_coding	protein_coding	-1.01147018887327	-2.01596443692218	1.46145152285429e-08	2.54931950017895e-05	Down in NR_Meteorin
Txndc12	 298370 	thioredoxin domain containing 12	protein_coding	protein_coding	-0.307314634100201	-1.23740231153541	0.000235363555538755	0.028003338811844	Down in NR_Meteorin
Tyrp1	 298182 	tyrosinase-related protein 1	protein_coding	protein_coding	-0.574486810303428	-1.48914765526338	0.00020899331227843	0.0267568960811513	Down in NR_Meteorin
Unc13b	 64830 	unc-13 homolog B	protein_coding	protein_coding	0.339888500552819	1.26565877308441	0.000556702589730816	0.0419358895341454	Up in NR_Meteorin
Usf3	 303946 	upstream transcription factor family member 3	NA	NA	0.421429964039017	1.33925433328189	1.27074172182392e-05	0.00422219064953639	Up in NR_Meteorin
Ush2a	 289369 	usherin	protein_coding	protein_coding	0.516274729488027	1.43025732142912	1.10816507450997e-05	0.00389236706509575	Up in NR_Meteorin
Usp2	 115771 	ubiquitin specific peptidase 2	protein_coding	protein_coding	0.388082286056075	1.30865271009751	0.00064663721731625	0.04425724491749	Up in NR_Meteorin
Vps13c	 363087 	vacuolar protein sorting 13 homolog C	protein_coding	protein_coding	0.444213656279343	1.36057233178292	0.000746337076994671	0.0490546740388774	Up in NR_Meteorin
Washc4	 314690 	WASH complex subunit 4	NA	NA	0.402180040418656	1.32150331172474	5.56853800301104e-05	0.0107929094211137	Up in NR_Meteorin
Wdfy3	 305164 	WD repeat and FYVE domain containing 3	protein_coding	protein_coding	0.389144704779684	1.309616773298	0.000178852182535442	0.024711705022595	Up in NR_Meteorin
Zbed5	 288622 	zinc finger, BED-type containing 5	protein_coding	protein_coding	-0.47665046700152	-1.39150921917845	0.000273912174316651	0.029151829094757	Down in NR_Meteorin
Zfc3h1	 314836 	zinc finger, C3H1-type containing	protein_coding	protein_coding	0.464578119111341	1.37991377234903	7.93128414423357e-05	0.0144606890542881	Up in NR_Meteorin
Zfhx3	 307829 	zinc finger homeobox 3	protein_coding	protein_coding	0.306444549328753	1.23665626314568	0.000623540170209931	0.0438313277377172	Up in NR_Meteorin
Zyg11b	 362559 	zyg-11 family member B, cell cycle regulator	protein_coding	protein_coding	0.297004096417176	1.22859046892507	0.000635106079099916	0.0440940563872603	Up in NR_Meteorin
